# Developing a mindfulness program for pre-clinical medical students in Indonesia: a mixed-methods study on suitability and appropriateness

**DOI:** 10.1186/s12909-025-07642-5

**Published:** 2025-07-17

**Authors:** Denish Gunawan, Lia Antico, William Nardi, Judson Brewer

**Affiliations:** 1 Brown University School of Public Health, Providence, RI USA; 2 Department of Behavioral and Social Sciences, Brown University School of Public Health, Providence, RI USA

**Keywords:** Mindfulness-based interventions (MBI), Medical students, Psychological distress, Program development, User-Centered design, Mixed methods research, Feasibility and acceptability

## Abstract

**Background & Aims:**

Medical students experience high rates of psychological distress, including depression, anxiety, burnout, and suicidality, due to rigorous academic demands. Mindfulness-Based Interventions (MBI) have demonstrated small-to-moderate effectiveness in reducing stress and improving well-being, yet culturally adapted and developmentally appropriate programs for Indonesian medical students remain limited. This study aims to develop and evaluate the Mindfulness Program for Pre-Clinical Medical Students in Indonesia (MPPMS-I) to determine its feasibility, acceptability, and suitability in addressing the mental health needs of pre-clinical medical students.

**Methods:**

This study employed a convergent mixed methods approach guided by the User-Centered Design (UCD) framework which emphasized iterative development based on user feedback. First-, second-, and third-year pre-clinical medical students were recruited between May and July 2024. The study was conducted in three iterative phases, each involving different participant groups to refine MPPMS-I: Phase 1 assessed initial impressions and interest, Phase 2 evaluated program relevance and clarity, and Phase 3 gathered detailed feedback on individual sessions to finalize the module. Quantitative analysis examined participant characteristics, interest levels, program relevance, applicability of learned concepts, and instructional clarity. Qualitative analysis were analyzed using framework analysis to explore themes such as participants’ first impressions, key lessons learned, beneficial aspects, daily integration of mindfulness practices, and barriers and facilitators to participation. Dual coding was used to enhance trustworthiness.

**Results:**

Findings revealed three key insights: (1) a moderate and rising level of interest (mean range: 5.75 to 6.6), supported by qualitative feedback indicating increased engagement following iterative refinement; (2) The program was perceived as highly relevant to students’ academic and personal challenges, particularly around stress, perfectionism, and communication; and (3) Participants reported a high likelihood of applying what they learned, such as mindfulness practices like S.T.O.P. meditation and journaling, into their daily routines.in their daily lives. Themes of relevance, practical benefit, and cultural fit emerged prominently in qualitative feedback.

**Conclusion:**

This study suggests that MPPMS-I is a feasible, culturally appropriate, and suitable intervention for pre-clinical medical students in Indonesia. Participants found it helpful for stress management, personal growth, and improving communication. As the program remains in an early evaluative phase, future research should include a longitudinal pilot study involving clinical students and assess long-term outcomes. Integrating MPPMS-I into medical curricula could offer a sustainable and context-sensitive approach to enhancing student well-being and resilience.

**Clinical trial number:**

Not applicable.

**Supplementary Information:**

The online version contains supplementary material available at 10.1186/s12909-025-07642-5.

## Introduction

Medical education is a highly demanding and stressful field, marked by rigorous academic standards, heavy workloads, and significant psychological pressure. These challenges can take a toll on students’ physical and mental well-being, increasing their risk of psychological distress—an umbrella term encompassing various common conditions such as depression, anxiety, burnout, stress, and suicidality—at higher rates than the general population [1–9]. Globally, the prevalence rates of depression, anxiety, and burnout among medical students were estimated to be 27%, 34%, and 33.4–55% respectively [10, 11]. According to a cross-sectional study conducted in 2021 across 49 medical faculties in Indonesia, the prevalence of depression and anxiety were 16.8% and 43.7% respectively [12]. Additionally, another cross-sectional study from 27 medical faculties in 2021 found that 35.5% of medical students experienced burnout [13]. Despite the numbers given, the rates of depression and anxiety in Asian countries are underreported compared to global standards due to the high stigma around mental health [14].

According to the Social Ecological Model of Health, stress among medical students arises from multiple, interacting levels: individual (e.g., low self-efficacy, maladaptive coping, low resilience, lack of self-care, financial strain, and certain personality traits), interpersonal (e.g., weak social support, low sense of belonging, and relational conflicts), organizational (e.g., high academic demands, competitive environments, and implicit medical norms), community (e.g., stigma toward mental health), and societal or policy-related factors (e.g., national medical education policies that overlook mental health support) [1, 4, 6, 15–25]. This framework underscores the importance of designing multi-level interventions to effectively address stress in medical students.

Several systematic reviews and meta-analyses have demonstrated that Mindfulness-based Interventions (MBI) can produce small-to-moderate effects in reducing stress and psychological distress symptoms in medical students [3, 7, 26–28]. Other reviews also mentioned that MBI can help in reducing anxiety, depression, and burnout [7, 11, 29]. Some other benefits of the MBI for medical students include enhancing self-awareness, resilience, empathy, self-compassion, altruism, and ability to focus [7, 30–32].

Despite the promising evidence of MBIs’ benefits, their use among university students in Indonesia remains limited. Some studies have investigated mindfulness breathing exercises [33] and culturally adapted internet-based mindfulness programs [34, 35]. This may in part reflect the relatively low awareness and concern for mental health issues among healthcare professionals in Indonesia, which can hinder the adoption of psychological interventions [36, 37]. Cultural adaptation is essential because Indonesia’s cultural values, based on Hofstede’s dimensions, emphasize hierarchy (high Power Distance), collectivism (low Individualism), long-term orientation, and restraint (low Indulgence), with moderate views on competitiveness (Masculinity) [38]. These cultural characteristics may shape how Indonesian students relate to mindfulness practices and determine how interventions should be designed for greater relevance and engagement.

Additionally, medical students face distinct challenges that may require further tailoring of intervention content. They tend to experience higher levels of psychological distress than peers in other fields [39] and often struggle to challenge authority [40]. A study has shown that Indonesian medical students differ slightly from other countries, particularly in showing higher Uncertainty Avoidance, which may lead to greater anxiety and discomfort in ambiguous situations [40]. These cultural and psychological factors highlight the need for mindfulness programs that are not only evidence-based but also tailored to the lived experiences and cultural context of Indonesian medical students. To date, however, no such program has been specifically developed.

To address this gap, the author proposes the development of a culturally adapted mindfulness-based mental health program designed specifically for pre-clinical medical students in Indonesia. This program, named the **Mindfulness Program for Pre-Clinical Medical Students in Indonesia (MPPMS-I)**, aims to address their mental health needs through primary and secondary prevention strategies. The development process was guided by the User-Centered Design (UCD) framework, an approach that emphasizes active user involvement throughout the design process [41, 42]. This proposed initiative, named the Mindfulness Program for Pre-clinical Medical Students in Indonesia (MPPMS-I), draws on the well-established Mindfulness-Based Stress Reduction (MBSR) program by Jon Kabat-Zinn [43]. It also incorporates insights from other mindfulness-based programs, such as the Unwinding Anxiety program by Judson Brewer [44] and the Mindfulness Self-Compassion program by Christopher K. Germer and Kristin Neff [45]. Together, these frameworks offer a holistic foundation for equipping medical students with practical mindfulness skills. In addition, the program was adapted to align with Indonesia’s cultural values and the multi-level stressors identified in the socioecological model. These adaptations aimed to ensure cultural relevance, psychological safety, and contextual fit. Further details on these adaptations are provided in the Methods section.

Finally, the MPPMS-I program aims to equip pre-clinical medical students in Indonesia with effective coping skills for stress management while fostering broader systemic changes that support mental health in academic and professional settings. This study elaborates on the development process of the MPPMS-I and critically evaluates its appropriateness and suitability in meeting the specific needs of this population. This evaluation ensures that the intervention is both feasible and impactful, aligning with the unique challenges faced by medical students and enhancing its integration into medical education.

## Methods

### Study design

**Fig. 1:**
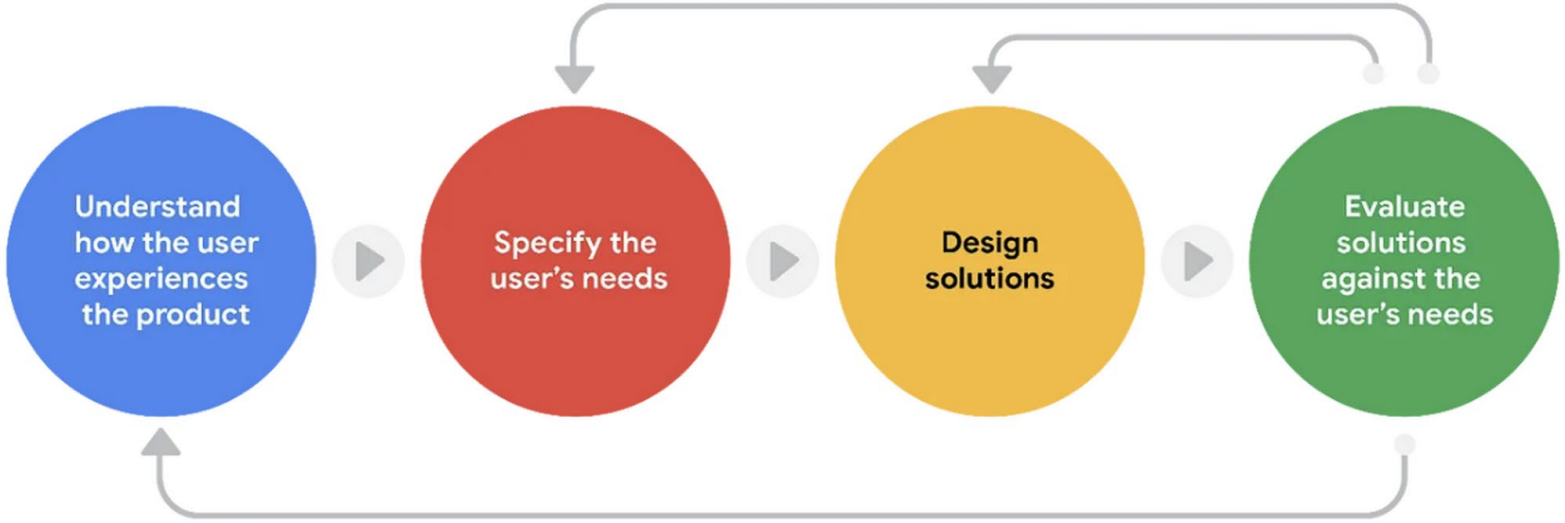
The User-Centered Design (UCD) Framework

The development of the MPPMS-I program was guided by the User-Centered Design (UCD) framework, an approach that emphasizes active user involvement throughout the design process [42]. This method ensures a comprehensive understanding of user experiences and needs while incorporating iterative design and evaluation phases [42, 46]. The UCD process involves four key steps: understanding user experiences, specifying user needs, designing solutions, and evaluating solutions based on user needs (as shown in Fig. 1) [42, 47].

### Program development process

Step 1: Understanding user experiences.

To identify medical students’ “pain points,” a targeted literature review was conducted using the Social Ecological Model of Health [48, 49], which categorizes stressors at individual, interpersonal, organizational, and societal levels. In addition, informal consultations with several medical students and faculty members were conducted to validate and contextualize these findings within the Indonesian medical education setting. These informal conversations were not part of formal data collection but served as preliminary insights to contextualize findings from the literature review. This process revealed common challenges faced by students, including academic pressure, emotional distress, limited support systems, difficulty in emotional regulation, and unfamiliarity with mindfulness-based practices.

Step 2: Specifying user needs.

Drawing from Step 1, the program development focused on addressing key individual and interpersonal needs. These included low self-efficacy, maladaptive coping strategies, challenges in emotional and stress regulation, and communication difficulties. A preliminary draft of the program was developed to target these areas, and was reviewed by a faculty member for early feedback. This input informed refinements to ensure the program was aligned with user concerns and expectations.

Step 3: Designing the intervention.

The design phase followed a **co-design approach**, consistent with the iterative nature of UCD. Medical students were involved beyond simple review; their input was integrated through informal consultations, review sessions, and early-stage feedback, which helped shape and refine the content, structure, and delivery format of the intervention.

The MPPMS-I program was developed as a seven-week mindfulness-based intervention, drawing from three evidence-based frameworks: Mindfulness-Based Stress Reduction (MBSR) by Jon Kabat-Zinn [43], Unwinding Anxiety by Judson Brewer [44], and Mindful Self-Compassion by Christopher Germer and Kristin Neff [45]. It also integrated insights from Lia Antico and Judson Brewer’s work on digital mindfulness training for healthcare professionals [50]. Each session was directly linked to the challenges identified earlier (see Table 1), and consisted of 65–75 min live sessions, paired with 15-minute daily home practices five days per week.

The program incorporated cultural adaptations to enhance its relevance for Indonesian students. It was delivered in a respectful, non-confrontational manner, aligning with Power Distance norms that emphasize deference to authority. Instructors were trained to foster psychologically safe spaces and use invitational language, allowing students to engage without feeling pressured to challenge hierarchy. Group-based activities such as sharing circles and compassionate listening were included to support collectivist values and foster a sense of community. To accommodate high uncertainty avoidance, the program offered structured weekly objectives and practical tools—such as the S.T.O.P. (S = Stop, T = Take a breath, O = Observe; P = Proceed) meditation and R.A.I.N. (R = Recognize; A = Acknowledge; I = Investigate; N = Note) meditation—that helped students reground themselves in the present moment during stressful situations and reduce anxiety.

The program also emphasized mindfulness as a discipline-oriented skill, supporting cultural norms around emotional restraint and self-regulation. Moral and relational values, such as kindness, responsibility, and harmony, were integrated into sessions addressing perfectionism, communication, and self-compassion. To reduce mental health stigma, the program used non-pathologizing language (e.g., “resilience” and “well-being”) and included private check-ins to enhance psychological safety. Designed to fit within the constraints of the academic calendar, the program was intended to be seen not as an added burden but as a support embedded within students’ existing schedules.

An important aspect of the design involved using life stories to contextualize mindfulness practices. In Phase 2, participants were invited to share their personal academic and emotional struggles in response to weekly themes; however, only partial examples were collected. In Phase 3, participants were encouraged more explicitly to contribute their stories, leading to a more complete and representative collection. These narratives generated by the students were then adapted into anonymized life stories used in the program’s final version. The aim was to enhance relatability by incorporating stories that reflected authentic and academically relevant challenges. These narratives were designed not only to illustrate mindfulness concepts but also to help future participants feel understood and supported by recognizing their own experiences within the stories.

Step 4: Evaluating the solution.

The final step involved evaluating the MPPMS-I program through a mixed-method design, integrated as part of the UCD feedback loop. The quantitative component involved descriptive analyses of participant characteristics, session evaluations (including relevance, clarity, and practical value), and student preferences regarding future delivery structure. The qualitative component explored participants’ experiences with the program, including perceived benefits, key lessons, application in daily life, challenges in participation, and suggestions for improvement. This evaluation provided insight into the program’s appropriateness, feasibility, and cultural fit for pre-clinical medical students in Indonesia. The finalized MPPMS-I module is provided in Supplementary Material 1.

**Table 1:** MPPMS-I program overview, pain points, and solutions in each session of the MPPMS-I

Program Overview		
Session	Pain Points	Solutions
Orientation	Lack of understanding of mindfulness	Understanding the concept of mindfulness
1. Mind-Body Connection	Not knowing oneself	Understanding how mindfulness can increase awareness that leads to self-discovery
	Disconnection between mind and body	Understanding the interplay between mind and body influencing each other
2. Perception and Responding to Stress	Lack of awareness of how perception influences stress levels	Understanding how perception influences stress levels
	Stressors and challenges in medical school, such as homework, reading materials, and exams that disrupt work-life balance	Differentiating between reacting to stress and responding to it mindfully
	Habitual reactions to stress	
3. How to Change Unhealthy Habits and Develop Healthy Habits	Unhealthy habits such as poor diet, lack of exercise, and inadequate sleep that affect well-being	Understanding habit loop
		Understanding the three gears to break free from unhealthy habit loops
		Understanding the importance of healthy habits
4. Perfectionism and Self-compassion	Stress from perfectionism which is related to performance anxiety, self-criticism, guilt, and shame	Understanding how perfectionism is related to stress and anxiety
	Empathy fatigue	Self-compassion as the antidote to perfectionism
5. Mindful Communication	Poor communication leads to misunderstanding and strained relationship	Mindful communication
6. Integrating Mindfulness into Daily Life	Difficulty in continuing mindfulness practices amidst the busy medical school life	Guidance on mindfulness practice continuation

### Instruments

The questionnaire used in all three phases was developed specifically for this study, guided by the User-Centered Design (UCD) framework. Its structure and item types were adapted from a related study by Antico and Brewer, which applied a UCD approach to develop a digital mindfulness training program for healthcare professionals [50]. Although the original supplementary instrument from that study was not publicly available, the study followed a similar structure and evaluative focus to assess participants’ perceptions of session clarity, relevance, usefulness, and overall engagement.

The instrument combined closed- and open-ended questions, including yes/no and sliding scale items (e.g., “How well does session [XX] meet your needs?” [0 = Not at all, 10 = Completely]) and reflective prompts (e.g., “What aspect of the session [XX] helped you the most as a medical student?”). The questionnaire was revised slightly across phases to match the evolution of the intervention.

The instrument was not formally validated or tested for reliability, as it was intended for formative use during the iterative development of the MPPMS-I program. Its primary function was to collect practical user feedback across cycles to refine the intervention content and structure. Formal validation is planned for future implementation, once the program materials and delivery format are finalized.

### Participants

The study was conducted in three phases between late May and early July 2024. Participants were recruited from first-, second-, and third-year pre-clinical medical students at the School of Medicine and Health Sciences, Atma Jaya Catholic University of Indonesia. Inclusion criteria included enrollment as a pre-clinical medical student in years one to three and any level of prior knowledge or familiarity with mindfulness practices. The exclusion criterion was students who declined to provide informed consent after randomization.

Participants were recruited using a two-stage process. A list of eligible students was obtained from the school administration, and a randomized sequence was generated using Randomizer.org [51]. From this list, male and female students across each academic year were systematically selected to ensure balanced representation. All selected students were contacted and provided with detailed information about the study, and written informed consent was obtained prior to participation. A stratified random sampling approach was then applied to finalize participant selection, balancing gender and year of study in each phase. Equal gender representation was intentionally maintained to minimize gender-related bias in qualitative feedback and to reflect evidence that male and female medical students may differ in psychological distress levels, coping styles, and responsiveness to mindfulness interventions [52, 53].

Each phase included a distinct set of participants, with no overlap between phases. In Phase 1, 12 participants were enrolled (6 males, 6 females); Phase 2 included 18 participants (9 males, 9 females); and Phase 3 involved 16 participants (8 males, 8 females). One male participant withdrew during Phase 3, resulting in a final count of 15 participants for that phase (7 males, 8 females). In total, 45 students participated across all phases of the study.

### Evaluation of appropriateness and suitability

The evaluation of appropriateness and suitability, corresponding to Steps 2, 3, and 4 of the UCD framework, was conducted in three iterative phases. While Step 1 was conducted separately, the data collection phases on these steps focused on specifying user needs, refining the intervention, and evaluating its appropriateness and suitability.

Each phase involved reviewing draft versions of the MPPMS-I program and completing a structured questionnaire (see Instruments section), distributed via Google Forms [52]. Successive phases built on the insights of previous ones, enabling systematic refinement of the intervention content, structure, and delivery method.

To give a clearer understanding of how each stage contributed to the program’s development and evaluation, Table 2 outlines the alignment of User-Centered Design (UCD) steps with the corresponding phases of the study, the types of data collected, and their contributions to the final intervention.

**Table 2:** Alignment of UCD steps with study phases, data collection methods, and development outcomes

Phase	UCD Step(s)	Quantitative Data	Qualitative Data	Purpose / Contribution to Development
Pre-phase	Step 1: Understand user experiences Step 2: Specify user needs	-	- Literature review on medical student stressors - Informal consultations with students and faculty	Informed the program’s conceptual focus and identified initial user challenges and contextual factors
Phase 1	Step 2: Specify user needs Step 3: Initial design testing	- Interest level ratings	- Firs impressions of program draft	Identified initial user expectations and reactions to draft content and structure
Phase 2	Step 2: Refine user needs Step 3: Refine prototype Step 4: Begin evaluation	- Session relevance - Clarity of home practice instructions - Usefulness of life stories	- Life stories - Reflections on key learning points - Helpful aspects - Preferences, barriers, facilitators	Guided structural revisions and refinement of session content, and life stories
Phase 3	Step 3: Finalize prototype Step 4: Evaluate solution	- Session relevance - Clarity of home practice instructions - Usefulness of life stories	- Life stories - Reflections on key learning points - Helpful aspects - Preferences, barriers, facilitators	Finalized content design, refined narratives, and confirmed cultural and contextual fit for target users

### Data analysis

#### Translation process

All responses collected via Google Forms were exported into Google Sheets and translated from Bahasa Indonesia to English using ChatGPT.com [55]. The translations were then reviewed by the author, who is fluent in both Bahasa Indonesia and English, to ensure accuracy and contextual fidelity before proceeding with data analysis.

#### Quantitative analysis

Quantitative data were analyzed descriptively using frequencies, means, standard deviations, and percentages in Google Sheets [56]. This analysis focused on participant characteristics, levels of interest in the program, evaluations of relevancy, application of learned lessons, instruction clarity, and participants’ preferences for the ideal program structure. Scores for interest levels, relevancy, application of learned lessons, and instruction clarity were categorized into three levels: low, medium, and high, while preferences for the ideal program structure were ranked as highly preferred, preferred, somewhat preferred, and least preferred (only in Phase 2 due to differences in response from participants).

#### Qualitative analysis

Qualitative data were analyzed using framework analysis, a method particularly suited for research with specific questions, predefined issues, and limited timeframes [57]. This approach allows for identifying commonalities and differences across participants, facilitating meaningful comparisons [58]. All qualitative data were organized, coded, and interpreted using Google Sheets to facilitate collaborative analysis and transparent mapping of themes [56]. The framework analysis followed six stages: familiarization, coding, developing a thematic framework, indexing, charting, and mapping and interpretation [58, 59]. Qualitative data focused on questions from three phases related to participants’ first impressions of the program, learned lessons, beneficial aspects of the program, daily integration of program elements, barriers and facilitators in participation, and feedback on the program.

During the qualitative analysis process, the author collaborated with a colleague, LA, who acted as the second coder to ensure consistency and rigor in the qualitative analysis. The analysis followed a systematic and structured approach, beginning with familiarization, during which both coders independently read the transcripts three times. This step allowed them to fully understand the content and context of the data while making initial notes. Next, the team developed a codebook using a combination of deductive and inductive approaches. Deductive codes were based on the questionnaire questions, while inductive codes emerged organically from the data as themes became apparent. To ensure consistency in coding, both coders independently analyzed the first three transcripts from three participants in Phase 1. This calibration exercise aligned their understanding of the codes and their application. Once the codes were established, they were grouped into categories to form an analytical framework. All responses were then double coded and indexed within this framework, ensuring reliability and thoroughness. The team proceeded to chart the data by summarizing responses for each code in a detailed spreadsheet created in Google Sheets. Each entry included a representative quotation to illustrate the themes identified. In the final stage, the team mapped and interpreted the data by identifying patterns and summarizing participants’ responses within each category. This systematic approach provided a comprehensive understanding of the qualitative data, enabling the identification of key insights and themes from participants’ experiences and perspectives.

Data saturation was monitored separately in each phase by observing when thematic repetition occurred and when no new categories or subthemes emerged from additional participant responses. Saturation was deemed achieved within a phase when responses from several participants no longer provided novel insights. Due to the phased design and the use of distinct participant groups in each phase, thematic saturation was assessed for each phase individually rather than across the entire dataset.

Furthermore, the analytical framework evolved over time to reflect the iterative refinement of the program. New codes were introduced in later phases to capture emerging themes, particularly regarding the clarity of session materials, the perceived cultural fit, and students’ emotional responses to specific content. These adaptations ensured that the analysis remained responsive to the program’s developmental trajectory and the contextual realities.

### Mixed-Method analysis

This study employed a convergent mixed-method design, in which quantitative and qualitative data were collected simultaneously but analyzed separately [60]. This approach provided a comprehensive understanding of participants’ perspectives by integrating numerical trends with in-depth qualitative insights [61]. Integration occurred through side-by-side comparison of results during interpretation. Quantitative patterns, such as high clarity or usefulness ratings, were explored in relation to qualitative feedback that explained why specific sessions or tools were impactful. Similarly, lower ratings were further examined through open-ended responses to identify content areas that required clarification or redesign. This process allowed for triangulation of data and enriched the interpretation of program effectiveness [60]. The integration of findings from both data types directly informed the iterative refinement of content, structure, and cultural presentation across the evolving drafts of the MPPMS-I program.

## Results

### Quantitative results

A total of 45 participants took part across three phases, with a nearly equal distribution of male and female students aged 18 to 22. Table 3 outlines the characteristics of the participants.

**Table 3:** Characteristics of participants

Characteristics	Phase 1 (*n* = 12)	Phase 2 (*n* = 18)	Phase 3 (*n* = 15)	Total (*n* = 45)
Gender Male, *n* (%) Female, *n* (%)	6 (50) 6 (50)	9 (50) 9 (50)	7 (46.7) 8 (53.3)	22 23
**Age**,** mean ± SD**	19.83 ± 1.03	19.89 ± 0.83	19.87 ± 0.99	
**Class Year n (%)** First Year Second Year Third Year	4 (33.3) 4 (33.3) 4 (33.3)	6 (33.3) 6 (33.3) 6 (33.3)	5 (33.3) 6 (40) 4 (26.7)	15 16 14

Note: n: frequency; SD: standard deviation; %: percentage

**The level of interest** in the MPPMS-I program was assessed across three phases, each with different participants, and categorized as low (1–4), medium (5–7), or high (8–10). Interest remained within the medium range across all three phases. Phase 1 had a mean score of 5.75 ± 1.36, Phase 2 was slightly lower at 5.2 ± 2.38, and Phase 3 had the highest score at 6.6 ± 1.55, nearing the upper limit of the medium category. The standard deviation in Phase 2 indicated greater variability in responses. Overall, the results demonstrate that as the program was refined from Phase 2 to Phase 3, the level of interest increased. (Table 4)

**Table 4:** Level of interest in the MPPMS-I program

Metric	Phase 1	Phase 2	Phase 3
Level of Interest mean **±** SD	5.75 **±** 1.36	5.2 **±** 2.38	6.6 **±** 1.55

Note: Note: SD: standard deviation

Table 5 shows the evaluation results of the MPPMS-I program’s effectiveness, including the relevance, likelihood of applying learned lessons, and clarity of instruction across seven sessions, divided into two distinct participant phases. Each category is reported with mean and standard deviation (SD) scores on a scale of 1 to 10. Scores were categorized into three levels: low (1–4), medium (5–7), and high (8–10). Notably, the categories “Life Story Usefulness” and “Home Practice Instruction’s Clarity” were not evaluated during the orientation session and session 6 due to the absence of life stories and home practice instructions.

The “Relevance to Personal Needs” was consistently rated high across all sessions for both Phase 2 (range: 7.06 to 8.06) and Phase 3 (range: 7.87 to 8.33), indicating that participants in both groups found all sessions to align well with their personal needs. Additionally, the “Relevance to Other Students’ Needs” was similarly rated high across all sessions for both Phase 2 (range: 7.33 to 8.00) and Phase 3 (range: 7.67 to 8.60), suggesting that participants believed all sessions effectively met the needs of other students.

The “Likelihood of Applying Learned Lessons” received ratings that ranged from medium to high. In Phase 2, the orientation session and Session 1 received medium ratings, with ratings of 6.94 and 6.89, respectively. In contrast, the remaining sessions in this phase received high ratings (range: 7.11 to 7.94). In Phase 3, all sessions were rated highly (range: 7.67 and 8.40). Although both phases displayed some variability in likelihood levels, the overall results suggest that participants generally felt confident about applying what they had learned.

The “Life Stories’ Usefulness” was consistently rated in the high category for both Phase 2 (range: 7.67 to 8.44) and Phase 3 (range: 8.07 to 8.47). The “In-Session Instructions’ Clarity” was uniformly rated high across all sessions in Phase 2 (range: 7.89 to 8.28) and Phase 3 (range: 8.20 to 8.67), indicating that participants found the in-session instructions clear and easy to follow. Lastly, the “Home Practice Instructions’ Clarity” was rated high across all sessions in both Phase 2 (range: 8.11 to 8.28) and Phase 3 (range: 8.00 to 8.63), suggesting that participants agreed on the clarity of the instructions for home practice.

In summary, participants in both Phase 2 and Phase 3 rated almost every session highly across all categories, indicating that all the program’s sessions were effective, relevant, applicable, and clear for all participants.

**Table 5:** Evaluation of MPPMS-I program relevance, application, and clarity across all sessions

Categories	Phase (mean ± SD)	Orientation Session	Session One	Session Two	Session Three	Session Four	Session Five	Session Six
**Relevance to Personal Needs Level**	Phase 2	7.33 ± 2.28	7.06 ± 2.39	8.00 ± 1.57	8.06 ± 1.73	7.78 ± 2.07	7.50 ± 2.15	7.22 ± 2.39
	Phase 3	7.53 ± 1.13	8.07 ± 1.28	7.87 ± 1.88	8.33 ± 1.45	8.20 ± 1.15	8.33 ± 1.40	8.07 ± 1.49
**Relevance to Other Students’ Needs Level**	Phase 2	7.33 ± 1.94	7.39 ± 2.03	7.78 ± 1.80	7.89 ± 1.68	7.83 ± 1.69	8.00 ± 1.85	7.56 ± 2.01
	Phase 3	7.67 ± 0.98	7.87 ± 1.30	8.13 ± 1.64	8.47 ± 1.25	8.27 ± 1.03	8.60 ± 1.40	8.53 ± 1.36
**Likelihood of Applying Learned Lessons Level**	Phase 2	6.94 ± 2.10	6.89 ± 2.52	7.50 ± 1.76	7.94 ± 1.70	7.39 ± 2.06	7.56 ± 2.20	7.11 ± 2.47
	Phase 3	7.67 ± 1.11	7.87 ± 1.46	8.13 ± 1.81	8.40 ± 1.55	8.27 ± 1.28	8.20 ± 1.42	8.40 ± 1.18
**Life Story’s Usefulness Level**	Phase 2	-	7.67 ± 2.11	8.44 ± 1.58	8.17 ± 1.69	8.33 ± 1.64	7.94 ± 1.86	-
	Phase 3	-	8.07 ± 1.49	8.07 ± 1.67	8.47 ± 1.41	8.40 ± 1.18	8.33 ± 1.50	-
**In-Session Instructions’ Clarity Level**	Phase 2	7.89 ± 1.60	8.17 ± 1.47	8.28 ± 1.56	8.17 ± 1.65	8.06 ± 1.70	8.11 ± 1.64	8.11 ± 1.68
	Phase 3	8.47 ± 1.30	8.40 ± 1.35	8.40 ± 1.80	8.67 ± 1.54	8.33 ± 1.23	8.20 ± 1.52	8.60 ± 1.30
**Home Practice Instructions’ Clarity Level**	Phase 2	-	8.17 ± 1.47	8.22 ± 1.73	8.11 ± 1.64	8.11 ± 1.75	8.28 ± 1.60	-
	Phase 3	-	8.00 ± 1.25	8.53 ± 1.64	8.33 ± 1.35	8.60 ± 1.30	8.53 ± 1.51	-

Note: SD: standard deviation

Table 5 presents participants’ preferences for the ideal structure of the MPPMS-I program in Phases 2 and 3. The table covers key program elements, including session pace, number of sessions, session duration, home practice duration, orientation session format, and group composition by rank. The rankings for Phase 2 and Phase 3 are presented using unified categories: Highly Preferred (Rank 1), Preferred (Rank 2), Somewhat Preferred (Rank 3), and Least Preferred (Rank 4, applicable only for Phase 2). An additional ranking has been included due to extra participant suggestions in Phase 2.

The findings show clear preferences across most categories. Most participants in both phases highly preferred one session per week, a total of five sessions, with each session lasting 60 min. For home practice, 15 min was the highly preferred duration. Regarding the orientation session, participants highly preferred holding it separately from the first session. Finally, most participants highly preferred being grouped with students from the same academic year, emphasizing a preference for year-specific group settings. These preferences provide a clear framework for structuring the program to meet participants’ needs effectively. (Table 6)

**Table 6:** Preferences for ideal program structure in phases 2 and 3

Category	Highly Preferred		Preferred		Somewhat Preferred		Least Preferred*
Phase	Phase 2	Phase 3	Phase 2	Phase 3	Phase 2	Phase 3	Phase 2
**Session’s Pace** **n (%)**	1 session/ week	1 session/ week	1 session/2 weeks	1 session/2 weeks	2 sessions /week	2 sessions/ week	1 session/ month
	*n* = 11 (61.1)	*n* = 8 (53.3)	*n* = 4 (22.2)	*n* = 4 (26.7)	*n* = 2 (11.1)	*n* = 3 (20)	*n* = 1 (5.6)
**Number of Sessions** **n (%)**	5 sessions	5 sessions	6 sessions	6 sessions	3 sessions	7 sessions	7 sessions
	*n* = 12 (66.7)	*n* = 9 (60)	*n* = 4 (22.2)	*n* = 4 (26.7)	*n* = 1 (5.6)	*n* = 2 (13.3)	*n* = 1 (5.6)
**Session’s Duration** **n (%)**	60 minutes	60 minutes	30 minutes	45 minutes	-	-	-
	*n* = 17 (94.4)	*n* = 13 (86.7)	*n* = 1 (5.6)	*n* = 2 (13.3%)			
**Home Practice Duration** **n (%)**	15 minutes	15 minutes	30 minutes	30 minutes	10 minutes	45 minutes	-
	*n* = 12 (66.7)	*n* = 8 (53.3)	*n* = 4 (22.2)	*n* = 5 (33.3)	*n* = 2 (11.1)	*n* = 2 (13.3)	
**Orientation Format** **n (%)**	Separate	Separate	Not separate	Not separate	-	-	-
	*n* = 13 (72.2)	*n* = 8 (53.3)	*n* = 5 (57.8)	*n* = 7 (46.7)			
**Group Composition** **n (%)**	Same year	Same year	Mixed year	Mixed year	-	-	-
	*n* = 15 (83.3)	*n* = 12 (80	*n* = 3 (16.7)	*n* = 3 (20)			

Note: n = frequency; *only applicable to Phase 2 due to expanded options

### Qualitative results

Seven themes were identified from participant feedback across the three phases of the study, guided by the structured codebook (see Supplementary Material 3). These include: (1) first impressions of the program, (2) lessons learned, (3) helpful aspects of the program, (4) daily integration of program elements, (5) barriers to participation, (6) facilitators of participation, and (7) suggestions for improvement. Theme 1 emerged from Phase 1, while Themes 2–7 were derived from Phases 2 and 3. A summary of all themes, subthemes, and categories across all phases is presented in Table 7.

### Theme 1: first impressions of the program

Participants expressed curiosity about mindfulness, with some describing it as unfamiliar. Despite this, many reported the content felt relevant and expressed interest in participating.

### Theme 2: lessons learned

Participants across both Phases 2 and 3 consistently described gaining key insights into mindfulness, stress regulation, and personal development. They demonstrated understanding of foundational mindfulness concepts, practical techniques such as S.T.O.P. and R.A.I.N. meditations, and their application in managing perfectionism, building healthy habits, and improving communication. Responses from both phases showed similar descriptions of the program’s key lessons and practices.

### Theme 3: beneficial aspects

Participants in both phases described the program as beneficial for emotional regulation, stress management, and developing healthier personal and academic habits. Phase 2 students additionally emphasized benefits related to self-care, academic performance, and empathy in clinical practice, while Phase 3 participants highlighted personal growth, the importance of self-compassion, and developing a present-focused mindset.

### Theme 4: daily integration of program elements

Students from both phases intended to apply mindfulness in their daily lives through intrapersonal practices like meditation and stress perception, as well as interpersonal skills such as mindful communication.

### Theme 5: barriers to participation

The most cited barrier across both phases was time constraints due to the academic workload. Some participants also reported low interest or motivation as challenges.

### Theme 6: facilitators of participation

Participants from both phases highlighted institutional support and credit incentives as key motivators. Phase 2 students emphasized the importance of curriculum integration, while Phase 3 students additionally noted that engaging content and voluntary participation would support enrollment and sustained interest.

### Theme 7: suggestions for modifications

Participants from both phases recommended content refinement. Phase 2 suggestions included more stress-related content specific to medical students and adapting materials to the Indonesian context. Phase 3 participants focused on improving instructional clarity and visual design.

**Table 7:** Summary of qualitative themes and representative quotes

Themes	Phase	Categories	Representative Quotes	
Theme 1. First Impressions of The Program				
Subtheme 1.1. Unfamiliar but interested	P1	-	*“Strange because it’s something new to me.”– P008“I was very interested after reading the modules and realized that the concept of mindfulness is more than just that.”– P004*	
Subtheme 1.2. Perceived relevance to student life	P1	-	*“Quite helpful so that I have another option to cope with stress during my studies.”– P012*	
**Theme 2. Learned Lessons**				
Subtheme 2.1. Mindfulness concepts and practices	P2 & 3	Foundational principles of mindfulness	*“… I learned about what mindfulness is*,* its benefits*,* and how to achieve it.”– P033 (Phase 3)*	
	P2 & 3	Mindfulness integration and continuing the practice	*“… integrate mindfulness into their daily lives… They reflect on their experiences during the program*,* write reflection journals*,* and receive guidance on how to continue mindfulness practice independently… introduced to choiceless awareness…”– P017 (Phase 2)*	
	P2 & 3	Mindfulness practices	*“… Using mindfulness techniques*,* such as walking meditation and STOP practice (Stop*,* Take a breath*,* Observe*,* Proceed)*,* students learn to change their perceptions and respond to stress…”– P029 (Phase 2)*	
Subtheme 2.2. Understanding the self	P2 & 3	Stress understanding	*“I learned to understand stress reactions and how I should respond to stress…”– P043 (Phase 3)*	
	P2 & 3	Mind-body connection	*“… provided a basic understanding of the mind-body connection…”– P044 (Phase 3)*	
	P2 & 3	Self-compassion as perfectionism’s antidote	*“I learned about perfectionism and self-compassion*,* where being too harsh on oneself is not good.”– P021 (Phase 2)*	
Subtheme 2.3. Personal growth	P2 & 3	Habit formation and change	*“… discusses the development of healthy habits… learn about the habit loop model*,* including the elements of triggers*,* behaviors*,* and consequences*,* and how to break the cycle of unhealthy habits using mindfulness….”– P029 (Phase 2)*	
	P2 & 3	Mindful communication	*“Learning about various communication styles and how I can be more mindful during conversations.”– P035 (Phase 3)*	
**Theme 3. Beneficial Aspects of the Program**				
Subtheme 3.1. Managing stress and improving well-being	P2 & 3	Managing academic and clinical stress	*“… relevant for medical students as it helps develop important skills needed in the medical field. The most helpful aspect is mindfulness practice… As a medical student facing heavy academic burdens*,* this ability is very useful for maintaining mental health and personal well-being.”– P015 (Phase 2)*	
	P2 & 3	Improving emotional regulation	*“… to be more organized*,* especially in managing emotions when facing something.”– P016 (Phase 2)*	
	P2 & 3	Sustaining mindfulness practice and integration of practice for long-term well-being	*“… this session is very valuable because it helps us learn how to apply what we have learned into daily life*,* thus improving ourselves in the future.”– P045 (Phase 3)*	
	P2	Balancing academic and personal life	*“… develop effective work-life balance”– P022 (Phase 2)*	
	P2	Enhancing self-care	*“Remembering to take care of oneself”– P014 (Phase 2)*	
	P3	Focusing on the present moment	*“The aspect of focusing on the present moment alone greatly helps me to release disappointment from the past and anxiety about the future*,* and just focus on what I can do now.”– P040 (Phase 3)*	
Subtheme 3.2. Fostering personal development and lifestyle changes	P2 & 3	Breaking free from bad habits	*“… breaking the bad cycle caused by stress.”– P035 (Phase 3)*	
	P2 & 3	Developing healthy habits	*“Must make it a habit to build a good lifestyle to remain healthy physically and mentally.”– P026 (Phase 2)*	
	P2 & 3	Developing self-compassion to combat perfectionism	*“… As a medical student*,* recognizing oneself as a perfectionist and practicing self-compassion can reduce stress and improve focus.”– P031 (Phase 3)*	
	P2	Improving academic performance and concentration	*“… to be more focused and better in pursuing education.”– P016 (Phase 2)*	
	P3	Accepting failure as a part of growth	*“… It helps in accepting failures and understanding that they are part of growth.”– P044 (Phase 3)*	
	P3	Supporting cognitive aspects	*“… offer cognitive*,* emotional*,* and physical benefits*,* especially for students who often feel anxious or cognitively impaired due to stress.”– P036 (Phase 3)*	
Subtheme 3.3. Fostering interpersonal relationships and communication	P2 & 3	Improving interpersonal relationships	*“… to foster kindness and better relationships while reducing stress.”– P029 (Phase 2)*	
	P2 & 3	Preventing misunderstandings	*“… Good communication skills are crucial in avoiding misunderstandings that can lead to stress and interpersonal problems in the medical education environment.”– P022 (Phase 2)*	
	P2	Fostering empathy and patient care*	*“… helps us develop skills to face patient suffering and interact effectively in clinical settings*,* build empathy*,* and enhance the quality of health services provided in the future.”– P024 (Phase 2)*	
**Theme 4. Daily Integration of Program Elements**				
Subtheme 4.1. Intrapersonal skills	P2 & 3	Integrating stress management strategies	*“Perception*,* responding to stress…”– P026 (Phase 2)*	
	P2 & 3	Shifting stress perception		
	P2 & 3	Practicing self-compassion	*“S.T.O.P. meditation*,* compassion*,* and the ability to change habits.”– P036 (Phase 3)*	
	P2 & 3	Changing habits		
	P2 & 3	Mindfulness practices	*“After completing the entire program*,* the element I want to integrate into my daily life is the mindfulness practice that I’ve learned I hope to take time every day for short meditation*,* breathing exercises*,* and practicing full awareness in daily…”– P015 (Phase 2)*	
Subtheme 4.2. Interpersonal skills	P2 & 3	Implementing mindful communication	*“… and elements of mindfulness in communication.”– P033 (Phase 3)*	
**Theme 5. Barriers to Program Participation**				
Subtheme 5.1.	P2 & 3	Time constraints	*“Some barriers… are the heavy schedule of classes and other activities. As medical students*,* our time is very limited*,* making commitment to the program regularly a challenge.”– P015 (Phase 2)*	
	P2 & 3	Lack of interest	*“Unpredictable schedule*,* other activities*,* upcoming exams*,* lack of interest” -P036 (Phase 3)*	
**Theme 6. Facilitators in Program Participation**				
Subtheme 6.1. Intrinsic factors	P2 & 3	Highlighting the program’s benefits	*“Awareness of the importance of mental health should be more emphasized as many students from my cohort skipped classes due to stress*,* fatigue*,* etc.”– P013 (Phase 2)*	
	P3	Engaging format	*“… short*,* interesting*,* and engaging sessions will increase student interest.”– P044 (Phase 3)*	
	P3	Voluntary participation	*“… students will be more motivated to participate if they are not pressured and required to attend… short*,* interesting*,* and engaging sessions will increase student interest.”– P044 (Phase 3)*	
Subtheme 6.2. Extrinsic factors	P2 & 3	Institutional support for the program	*“Support from the faculty and university would be helpful. If the program is officially recommended or supported by the institution*,* it will be easier for students to get involved… if the program can offer credits that count towards academic assessments*,* it will encourage students to participate.”– P015 (Phase 2)*	
	P2 & 3	Offering credit as an incentive	*“… the program can offer credits that count towards academic assessments*,* it will encourage students to participate.”– P015 (Phase 2)*	
	P2	Integrating the program into the curriculum	*“If it offers grades/joining a block system”– P026 (Phase 2)*	
**Theme 7. Suggestions for Modifications to The Program**				
Subtheme 7.1. Refining the content	P2 & 3	Adding detailed explanations of the image in the modules*	*“I hope there will be more explanations at the bottom of the images.”– P013 (Phase 2)*	
	P2	Including relevant content about stress in medical students*	*“… perhaps a session on stress and anxiety management commonly faced by medical students could be added… a session on how to apply mindfulness practices in a clinical context… would also be very beneficial… it might be worth considering including topics on self-care and preventing burnout in the medical profession.”– P015 (Phase 2)*	
	P2	Considering the cultural contexts of Indonesian medical students*	*“The importance of adapting mindfulness programs to the cultural and environmental context of medical students in Indonesia.”– P023 (Phase 2)*	
	P3	Refining instructions’ clarity	*“… Maybe instructions for home practice could be written more clearly and completely*,* and the modules could be designed more attractively.”– P036 (Phase 3)*	
	P3	Revamping the modules design		

## Discussion

This study aimed to develop and evaluate the MPPMS-I program as culturally appropriate and suitable for Indonesian pre-clinical medical students. A total of 45 participants took part across three phases, with balanced gender, and class-year representation,. Quantitative results showed a medium level of interest in the MPPMS-I program across all phases (mean range: 5.75 to 6.6), increasing with program refinement. The effectiveness evaluation in Phases 2–3 demonstrated consistently high scores across seven sessions, except two medium scores for orientation and Session 1 in Phase 2. The evaluation assessed relevance, applicability, clarity, and home practice guidance. Participants expressed a preference for one weekly session instead of five sessions lasting 60 min each, along with 15 min of daily home practice, a standalone orientation, and year-specific grouping.

Qualitative results highlighted several key themes across the three phases: (1) **first impressions of the program**, including a feeling of unfamiliarity with mindfulness, but there is a growing interest and recognition of the program’s relevance; (2) **lessons learned**, which encompassed mindfulness concepts and practices, self-understanding, and personal growth; (3) **beneficial aspects of the program**, such as stress management, well-being improvement, personal development, lifestyle changes, and enhanced interpersonal relationships and communication; (4) **daily integration of program elements**, including stress management strategies, shifting stress perception, practicing self-compassion, changing habits, mindfulness practices, and mindful communication; (5) **barriers to participation**, primarily time constraints and lack of interest; (6) **facilitators of participation**, such as emphasizing the program’s benefits, providing an engaging format, ensuring voluntary participation, institutional support, offering academic credits, and integrating the program into the curriculum; and (7) **suggestions for modifications**, including refining content and considering the cultural context of Indonesian medical students.

### Integration of quantitative and qualitative findings

By combining numerical trends with students’ reflections, three core conclusions emerged. First, participants expressed interest in the MPPMS-I program, as indicated by medium interest levels across all phases and qualitative impressions of curiosity and engagement. The rise in interest from Phase 2 to Phase 3 likely reflects the impact of iterative refinements that improved the program’s clarity, relevance, and structure. Second, students perceived the content as highly relevant to their experiences in medical education, particularly in sessions focused on stress perception, perfectionism, and mindful communication. This was evident in the consistently high relevance ratings observed in Phases 2 and 3. These topics likely resonated due to culturally specific academic pressures, internalized expectations for high achievement, and relational dynamics influenced by Indonesia’s cultural context [1, 4, 17]. Such characteristics are common among Indonesian medical students, particularly those associated with a high Power Distance and high Uncertainty Avoidance cultural profile [40]. Developing curricula that enhance medical students’ resilience by incorporating self-care techniques in stressful situations may help reduce burnout, supporting the importance of the mindfulness-based themes emphasized in this program [62]. Moreover, as the program became more contextualized using relatable life stories, clearer instructions, and enhanced visual design, students were better able to connect with the material. This process also demonstrates the effectiveness of the iterative User-Centered Design (UCD) approach used in developing the program. Third, participants expressed an intention to integrate specific practices such as mindful breathing, the S.T.O.P. meditation, and journaling into their daily lives, corroborated by high ratings for the likelihood of applying learned lessons. Together, these patterns indicate that students found the MPPMS-I program relevant, practical, and well-suited to support their well-being in the context of medical education.

### Balancing preferences and pedagogical needs

The program structure preferences found in this study align with existing mindfulness-based interventions (MBIs) for medical students, which typically span 6–10 weeks (this study: five weeks) and have session durations of 60–90 min (this study: 60 min) [28, 63–66]. Research suggests that shorter interventions can still provide significant benefits while improving accessibility, particularly by addressing time constraints—one of the main barriers to participation in this study [8, 28, 31]. Although participants expressed a preference for a five-week program with 60-minute sessions and 15 min of home practice, the author opted for a seven-week structure with sessions lasting 65 to 75 min, maintaining the 15-minute home practice. This decision was made to accommodate an additional orientation session and a concluding session (session six) focused on summarizing key concepts and integrating mindfulness into daily life. Extending both the number and duration of sessions ensured that all material was covered at a steady, manageable pace.

The findings regarding barriers and facilitating factors in program participation were consistent with previous studies and further contextualize the structural preferences described above. Time constraints emerged as a significant barrier, as highlighted in several Randomized Controlled Trials (RCTs), which cited challenges in time management, the demanding nature of medical education, and a lack of control over schedules [8, 30]. However, a study acknowledged that time constraints will always exist since there may never be a “right time” for self-care within medical training due to systemic issues in medical education [67]. Flexible scheduling and shorter interventions have been suggested as potential solutions [8, 28, 31, 62, 68]. Another barrier identified was the lack of interest, which could be attributed to skepticism about mindfulness, including concerns about it being perceived as pseudoscience and doubts about its evidence-based benefits [8]. A key strategy to overcome this skepticism is linking mindfulness to its clinical applications and emphasizing its scientific foundation, which aligns with one of the facilitating factors mentioned in this study—highlighting practical benefits to increase student interest [68]. Institutional support was another crucial factor in facilitating the process. Studies have consistently shown that integrating mindfulness into the core medical school curriculum encourages participation because it eliminates the need for additional time commitments [29, 31, 63, 68]. Lastly, voluntary participation was identified as an essential factor in engagement. Multiple studies emphasized that mindfulness practice cannot be forced and requires an open attitude to be effective [63, 64, 67, 68]. One systematic review further revealed that students who chose to participate voluntarily reported higher satisfaction levels than those in mandatory mindfulness programs [56].

### Theoretical and cultural alignment

The development and refinement of the MPPMS-I program were informed by two key frameworks: the Social Ecological Model and the User-Centered Design (UCD) approach. The Social Ecological Model highlights how stress arises from interacting influences at individual, interpersonal, and institutional levels—such as maladaptive coping, relational tension, and academic pressure—especially prevalent among Indonesian medical students [1, 4, 6, 15–25]. These multilevel stressors were directly addressed through session content on self-compassion, mindful communication, and stress perception, as reflected in participants’ feedback and integration of these tools into daily life.

Meanwhile, the UCD framework guided the program’s iterative development by actively involving students in shaping content, structure, and delivery [42]. Their feedback directly informed several refinements, including the integration of relatable life stories, the use of secular and accessible language, and adjustments to session pacing and instructional clarity. These modifications enhanced the program’s cultural relevance and accessibility while also illustrating the effective use of UCD principles in designing mindfulness interventions for medical education.

Participants’ recommendations on adapting the program to Indonesian cultural contexts align with the rationale provided by Loucks et al. and Listiyandini et al., who emphasize the importance of community input when designing mindfulness programs in the community [35, 69]. The cultural adaptations made in this study included using the Indonesian language as the primary mode of delivery and employing secular terminology to enhance acceptability while still respecting the Buddhist roots of mindfulness [70, 71]. These adaptations aligned with Hofstede’s cultural dimensions for Indonesia’s medical students, including high power distance, collectivism, and high uncertainty avoidance [38, 40]. These cultural traits likely contributed to participants’ preference for structured guidance, psychologically safe group formats, and teacher-led delivery.

Comparable findings were observed in Malaysia, a country with cultural characteristics similar to Indonesia. A study reported that Malaysian medical students were more receptive to mindfulness programs that were religiously neutral, scientifically grounded, and presented in a structured and practical manner [31]. Given Malaysia’s similarly high power distance, collectivist orientation, and high uncertainty avoidance, this parallel reinforces the importance of local adaptation to ensure engagement and reduce resistance in collectivist Southeast Asian contexts [40].

Aligning the program design with both theoretical models and cultural context was crucial to ensure that the MPPMS-I program was not only developmentally appropriate but also culturally sensitive and pedagogically sound for Indonesian medical students.

### Strengths and limitations

This study offers a distinct contribution to the mindfulness and medical education literature by being, to the best of the author’s knowledge, the first to apply a User-Centered Design (UCD) framework to develop a Mindfulness-Based Intervention (MBI) specifically for medical students. While most existing studies focus on evaluating feasibility or pilot outcomes, few detail the iterative design process and direct involvement of students in shaping program content, delivery, and cultural adaptation. By documenting the full development cycle, this study addresses a critical gap in user-driven program design for MBIs in medical education.

One of the strengths of this study lies in its mixed-methods design, which allowed for an in-depth exploration of both the measurable outcomes and lived experiences of participants. The integration of quantitative findings on session relevance and applicability with rich qualitative insights into students’ reflections provided a well-rounded understanding of the program’s perceived impact. Moreover, the use of iterative feedback loops throughout the three phases demonstrated the practicality and responsiveness of the UCD framework in shaping interventions that are both pedagogically sound and culturally attuned. Additionally, the use of dual coding during qualitative analysis enhanced trustworthiness and reduced interpretive bias, further strengthening the study’s methodological rigor.

From a practical standpoint, the MPPMS-I program addresses a significant and often overlooked need in Indonesian medical education. It provides critical knowledge on how culturally adapted and structured mindfulness programs can equip students with essential coping mechanisms to manage academic and professional stress. As the burden of burnout and stigma surrounding mental health persists, this study offers an actionable model for program development in similar high-stress academic environments. Given the growing emphasis on mental health and well-being in medical education, these findings provide a strong foundation for institutions to consider piloting or integrating mindfulness-based training into their curricula.

Despite its strengths, this study also has several limitations. These are grouped thematically below for clarity.

### Design limitations

One key methodological constraint occurred during the early stages of the UCD process. The identification of medical students’ “pain points” and potential solutions was based primarily on a targeted literature review rather than formal input-gathering methods such as surveys or focus groups. However, this was partially mitigated by informal consultations with medical students and faculty members, which helped contextualize and validate the findings in the Indonesian academic environment. While this approach ensured relevance in the initial design, future studies could strengthen this step by incorporating structured input from users earlier in the development process to enhance alignment with students’ lived experiences.

### Data quality limitations

In the qualitative analysis, some inconsistencies were noted. One data point was missing, one participant repeated the same answers across multiple questions, and another appeared to misplace responses between sessions. Despite these issues, data saturation was achieved within each phase, and two coders were involved in the analysis to increase reliability and interpretive rigor.

### Timing and response Bias

The study was conducted during students’ examination period, which may have limited the attention participants could give to reflective questions or program engagement. However, the fact that students still participated despite this context highlights the perceived value of the program. Clear instructions and generous time allocations were provided to mitigate potential response bias.

### Generalizability

This study focused solely on pre-clinical medical students in Indonesia. Clinical students—who experience different schedules and stressors due to hospital rotations—were not included. Given these contextual differences, the findings may not be directly transferable to all stages of medical education. Future research should expand to include clinical cohorts to better understand the program’s relevance across the medical training continuum.

### Delivery format preferences

The study did not explore preferences for online versus in-person program delivery. As hybrid learning becomes more common, future research should investigate how different delivery formats influence engagement and outcomes.

### Instrument formatting limitations

Lastly, variations in open-text responses within the questionnaire—particularly in questions on preferred program structure—made it difficult to fully standardize these results. Although this offered rich qualitative data, a more consistent format might yield clearer preferences.

### Future directions

Looking ahead, the next logical step is to conduct a longitudinal pilot study to further assess the acceptability, feasibility, and long-term effectiveness of the MPPMS-I program among pre-clinical medical students in Indonesia. This would provide the necessary empirical validation to support its integration into formal medical school curricula. The current version of the MPPMS-I serves as a foundational framework that can be further refined and adapted to meet the unique needs of clinical students, who face distinct stressors during hospital-based training. Sustaining the program’s relevance and impact will require institutional support, flexible delivery formats, and ongoing adjustments informed by student feedback. Ultimately, this study lays important groundwork for future research and implementation, supporting a more structured and culturally responsive approach to mindfulness education within medical training.

## Conclusion

This study demonstrated that the MPPMS-I program is a feasible, culturally appropriate, and suitable intervention for pre-clinical medical students in Indonesia. Participants expressed interest in the program and found it helpful in managing stress, personal growth, and interpersonal communication. While these findings highlight the program’s promise, further refinement is necessary before full implementation. A longitudinal pilot study is a crucial next step to evaluate its acceptability, feasibility, and long-term effectiveness among both pre-clinical and clinical cohorts. Given the growing recognition of mindfulness-based interventions (MBIs) in medical education, integrating MPPMS-I into medical school curricula could offer a sustainable and contextually grounded approach to enhancing student resilience and mental well-being. Long-term success will require institutional support, flexible delivery formats, and continued adaptation informed by user feedback.

## Electronic supplementary material

Below is the link to the electronic supplementary material.

Mindfulness Program for Pre-Clinical Medical Students in Indonesia (MPPMS-I) - Curriculum and Teaching Guide

Mindfulness Program for Pre-Clinical Medical Students in Indonesia (MPPMS-I) Modules Development Questionnaires

Codebook

## Data Availability

The datasets used and analyzed during the current study are available from the corresponding author upon reasonable request.

## Abbreviations

MBI Mindfulness: Based Interventions
MPPMS: I Mindfulness Program for Pre-Clinical Medical Students in Indonesia
UCD User: Centered Design
