# Supplementary material for: Developing a mindfulness program for pre-clinical medical students in Indonesia: a mixed-methods study on suitability and appropriateness
Source: BMC Med Educ. 2025 Jul 17;25:1072. doi: 10.1186/s12909-025-07642-5 (PMC12272978; doi:10.1186/s12909-025-07642-5)
Supplement: Supplementary file 1 — Mindfulness Program for Pre-Clinical Medical Students in Indonesia (MPPMS-I) - Curriculum and Teaching Guide [file 12909_2025_7642_MOESM1_ESM.docx]

**Mindfulness Program for Pre-Clinical Medical Students in Indonesia (MPPMS-I) Modules Development Questionnaires**

**Introduction:**

Thank you for participating in our 7-week mindfulness-based program for pre-clinical medical students’ modules development questionnaires. Your feedback is essential to help us improve and tailor the program to better meet your needs. Please take a few minutes to answer the following questions.

Here are the Phase 1 questionnaires to gauge your interest in this module.

**Phase 1: Initial Impressions**

**Open Question:**

1. **Code Number:**

Please enter the code number that you received from the email.

**Closed Question:**

1. **Program Interest:**

After reading the introductory module, would you be interested in attending the program if it is offered to you?

- 1. Yes
  2. No

**Slider Questions**

1. **Interest Level:**

How interested are you in attending this program? (0 = Not interested at all, 10 = Extremely interested)

**Open Question:**

1. **First Impression:**

What is your first impression after reading the introductory module?

**Phase 2 & 3:**

**Introduction:**

Thank you for participating in our 6-week mindfulness-based program for preclinical medical students’ module development questionnaires. Your feedback is essential to help us improve and tailor the program to better meet your needs. Please take a few minutes to answer the following questions.

Here are the Phase 2 & 3 questionnaires to gather your feedback on this module.

There are 2 main sections in these questionnaires:

1. Feedback on each session (consisting of 7 sessions, from the orientation session to the sixth session)
2. Overall program feedback

Please answer all the questions carefully.

**Each Session Feedback**

**Open Questions:**

1. **Code Number:**

Please enter the code number that you received from the email.

1. **Learning Reflection:**

Please explain in your own words what you have learned in session [XX].

1. **Medical Student Relevance:**

What aspect of session [XX] helped you the most as a medical student?

1. **Future Doctor Relevance:**

What aspect of session [XX] helped you the most as a future doctor?

1. **Personal Life Relevance:**

What aspect of session [XX] helped you the most in your personal life?

**Slider Questions (0-10):**

1. **Session Needs:**

How well does session [XX] meet your needs? (0 = Not at all, 10 = Completely)

1. **Medical Student Needs:**

How well does session [XX] meet the needs of other medical students you know? (0 = Not at all, 10 = Completely)

1. **Practical Application:**

How likely are you to put what you have learned in session [XX] into practice? (0 = Not at all likely, 10 = Very likely)

1. **Story Usefulness:**

How useful are the real-life stories described in session [XX]? (0 = Not useful at all, 10 = Extremely useful)

1. **In-Session Instructions Clarity:**

How clear are the instructions for in-session practices in session [XX]? (0 = Not clear at all, 10 = Very clear)

1. **Home Practice Instructions Clarity:**

How clear are the instructions for home practices in session [XX]? (0 = Not clear at all, 10 = Very clear)

**Open Questions:**

1. **Additional Stories:**

Do you have other better real-life stories that came to mind related to session [XX]? If so, please share them. If not, please write "I do not."

1. **Understanding Home Practices:**

Have you had any confusion or difficulties in understanding home practices in session [XX]? If so, please describe them. If not, please write "I do not."

1. **Performing Home Practices:**

Have you had any difficulties in doing home practices in session [XX]? If so, please describe them. If not, please write "I do not."

**Overall Program Feedback**

**Multiple-Choice Questions + Open Questions:**

1. **Session Pace:**

According to your experience, what is the best pace for the sessions we should suggest to users?

- - 1. One session per week
    2. One session every two weeks
    3. Two sessions per week
    4. d. Other ____

1. **Number of Sessions:**

According to your experience, how many sessions should we suggest to users?

- - 1. 5 sessions
    2. 6 sessions
    3. 7 sessions
    4. Other ____

1. **Session Length:**

According to your experience, what is the best length for each session we should suggest to users?

- - 1. 60 mins
    2. 90 mins
    3. 120 mins
    4. Other ____

1. **Home Practice Length:**

According to your experience, what is the best length for home practices we should suggest to users?

- - 1. 15 mins
    2. 30 mins
    3. 45 mins
    4. Other ____

1. **Orientation Session:**

According to your experience, should the orientation session be separated or integrated into the first session?

- - 1. Orientation session should be separated
    2. Orientation session should be integrated into the first session (If you choose this, continue to question 20)

1. **First Session Length:**

If the orientation session is integrated into session one, would it be okay if the first session is longer than other sessions?

- - 1. Yes
    2. No
    3. If yes, what is the maximum additional length of time? _____

1. **Participation Preference:**

According to your experience, would you prefer to participate with students from the same year or combined years?

- - 1. Same year
    2. Combined years

**Reordering:**

1. **Session order preference:**

Please reorder the following six modules according to your preferences. Simply drag and drop each module into your preferred order.

**Closed questions**

1. **Program Interest:**

After reading the introductory module, would you be interested in attending the program if it is offered to you?

- 1. Yes
  2. No

**Slider Questions**

1. **Interest Level:**

How interested are you in attending this program? (0 = Not interested at all, 10 = Extremely interested)

**Open Questions:**

1. **Daily Life Integration:**

After completing the entire program, what elements do you want to integrate into your daily life?

1. **New Content Suggestions:**

Do you have any suggestions for new content to include in the program?

1. **Barriers to Participation:**

What barriers might medical students face in joining this program?

1. **Facilitators to Participation:**

What factors do you think would facilitate medical students joining this program?

1. **Additional Feedback:**

Do you have any other comments, questions, or feedback to help us improve this program?

Thank you for your valuable feedback!

This questionnaire should help us gather detailed feedback to refine and improve The Mindfulness-based Program for Pre-Clinical Medical Students.
