# Supplementary material for: Developing a mindfulness program for pre-clinical medical students in Indonesia: a mixed-methods study on suitability and appropriateness
Source: BMC Med Educ. 2025 Jul 17;25:1072. doi: 10.1186/s12909-025-07642-5 (PMC12272978; doi:10.1186/s12909-025-07642-5)
Supplement: Supplementary file 2 — Mindfulness Program for Pre-Clinical Medical Students in Indonesia (MPPMS-I) Modules Development Questionnaires [file 12909_2025_7642_MOESM2_ESM.docx]

**CODEBOOK**

| P | S | Code | Type | Brief Definition | Definition | Inclusion Criteria | Exclusion Criteria | Example | Example Description |
| --- | --- | --- | --- | --- | --- | --- | --- | --- | --- |
| 1 |  | First impression | Deductive | Initial thoughts and feelings about the program | Describes the participant's initial thoughts and feelings after reading the overview of the program | Includes participant's reactions, expectations, or emotional response to the initial description of the program | Excludes any reflection related to specific learning or perceived benefits from the program. | "My first impression when reading this introductory module was quite familiar because it reminded me of myself, who these days has been feeling quite stressed preparing for exams..." | Participant describes their first impression of the program in relation to the current overwhelming school life and finds the program either interesting or strange. |
| 2 |  | Learned lessons | Deductive | Insights on materials and techniques learned from the program | Refers to particular materials or experiential practices, including mindfulness practices, mindfulness exercises, and mindfulness techniques that the participant learned from reviewing the program. | Includes specific mentions of materials, mindfulness practices (such as sitting meditation, yoga, walking meditation), mindfulness exercises (such as the 9 dots exercise, habit loop exercise), and mindfulness techniques (such as S.T.O.P. or R.A.I.N. meditation). | Excludes general reflections that do not reference specific practices or techniques. | "I learned about the types of stress and how to overcome them through mindfulness." | Participant describes how perception affects stress levels and the importance of S.T.O.P. meditation during stressful times. |
|  | a | Learned information | Inductive | Mindfulness facts learned from program | Refers to any mindfulness information or facts the participant has learned from the program's modules. | Includes any information about the program's materials such as what mindfulness is and the body's physiological response to stress and mindfulness practices. | Excludes general reflections that do not reference specific skills, practices, or techniques. | "I learned that the mind and body are interconnected, where if our mind feels panic or anxiety, the body will react with stress." | Participant learned about the body's reponse to stress as well and the interconnected nature of physical and mental stress. |
|  | b | Learned skills | Inductive | Mindfulness skills learned from program | Refers to any mindfulness skills, techniques, or practices the participant may have learned to perform from the program's modules. | Includes mindfulness practices (such as sitting meditation, yoga, walking meditation, mindful communication), mindfulness exercises (such as the 9 dots exercise, habit loop exercise), and mindfulness techniques (such as S.T.O.P. or R.A.I.N. meditation). | Excludes general reflections that do not reference specific information. | " Practicing RAIN meditation to manage stress and the demands of medical education more effectively." | Participant describes how the learned a helpul mindfulness technique called RAIN meditation |
| 3 |  | Prior misconceptions | Inductive | False belief about the program or mindfulness practices | Refers to false or untrue beliefs about mindfulness/mindfulness practices that a participant may have had prior to engaging with this program. | Includes uninformed or incomplete definitions of what mindfulness is or how it may be used | Excludes correct definitions | " I used to think mindfulness was about clearing and calming the mind, but after reading this, I realized that mindfulness means focusing on one issue and acknowledging and accepting the mistakes we've made." | Participant acknowleges that prior to engaging with the program, they did not have a full understanding of mindfulness and recognizes how the program improved their understanding. |
| 4 |  | Helpful aspects of the program | Inductive | Perceived general usefulness of the program | Refers to participants' overall views on the program's benefits and value. | Includes references to how the program content supports personal growth, academic success, or emotional health. | Excludes any statements that do not directly relate to perceived usefulness or value of the program. | "In reality, mindfulness is very useful in all aspects of life to help us live more focused and well." | Participant mentions that mindfulness is useful in helping people to live more focused and well. |
|  | a | Helpful aspects for medical students | Deductive | Perceived usefulness of the program in medical training | Refers to participant's thoughts, insights, and takeaways about how the program can be helpful in their journey as a medical student. | Includes references to how the program content supports personal growth, academic performance, or emotional well-being as a medical student. | Excludes general comments on personal development that do not explicitly link to the medical student. | "Recognizing and accepting one’s own shortcomings is a valuable lesson ... to prevent dissatisfaction and burnout." | Participant mentions that self-compassion is crucial to avoid being overly critical and accepting failures, knowing that failure is a part of growth. |
| 5 |  | Home practice understanding challenges | Deductive | Confusion or difficulties with home practices | Refers to any confusion of difficulties in understanding the home practices of the program. | Includes any confusion, misunderstanding, or lack of clarity regarding the program's home practices. | Excludes comments about understanding program content unrelated to home practices. | "... I don't understand mindful eating (does it mean mindful eating to pay attention to what we eat? or behavioral?)." | Participant mentions about having difficulty in understanding about the home practices in a certain session. |
| 6 |  | Daily integration of program elements | Deductive | Future application of program techniques in daily life | Refers to particular materials or experiential practices, including mindfulness practices, mindfulness exercises, and mindfulness techniques from the program that the participant plans to include in their daily life. | Includes specific examples of how participants plan to incorporate program elements into daily routines, including mindfulness practices (such as sitting meditation, yoga, and walking meditation), mindfulness exercises (like the 9 dots exercise and habit loop exercise), and mindfulness techniques (such as S.T.O.P. or R.A.I.N. meditation). | Excludes ambiguous intentions or plans that are not directly related to program content. | "Communication with greater awareness and understanding of others to avoid misunderstandings." | Participant mentions wanting to apply good communication skills to avoid misunderstanding. |
| 7 |  | Barriers in participating | Deductive | Factors impeding participation | Descriptions of impeding factors that might prevent the participant from joining the program. | Includes any mention of obstacles such as time, scheduling conflicts, or external pressures that hinder participation. | Excludes factors not directly affecting the participant's ability and motivation to participate in the program. | "Unpredictable schedule, other activities, upcoming exams, lack of interest" | Participant mentions that time constraints caused by multiple commitments are hindering their participation in the program. |
| 8 |  | Facilitating factors in participating | Deductive | Factors encouraging participation | Descriptions of supporting factors that encourage the participant to participate in the program. | Includes any references to external or internal motivators that encourage program participation. | Excludes irrelevant facilitators unrelated to the program. | "Coordination with the faculty to get an ideal schedule, incentives, opportunities to build good relationships with faculty/program facilitators, presentation of benefits that are good and relevant to medical students" | Participant mentions that the program being officially supported by the institution and offering credit would make it more interesting to join. |
| 9 |  | General feedback or comments | Deductive | Feedback or suggestions for the program's content | Mentions of any additional thoughts, feedback, comments, or new content suggestion that the participant offers after interacting with the program's content. | Includes any kind of feedback or additional comments not directly captured by other codes. | Excludes comments or complaints unrelated to the program or that don't include suggestions for improvement. | "How to maintain and apply self-discipline so that what you learn is not only done in the near future" | Participant suggests that the program would be more effective if it focused on emotional management and was delivered interactively to a small group of students. |
|  | a | Constructive feedback | Inductive | Critical comments aimed at improving the program | Any feedback that points out areas for improvement, offering suggestions or critiques about the program's structure, delivery, or content. | Includes suggestions for improvements, critiques, or requests for changes in the program. | Excludes purely negative feedback without suggestions for improvement or unrelated comments. | "The program is good. Maybe instructions for home practice could be written more clearly and completely, and the module could be designed more attractively." | Participant offers feedback about program length and suggests adding more interactive components for engagement. |
|  | b | Positive feedback | Inductive | Affirmations or praise about the program | Any comments that express approval or satisfaction with the program's content, delivery, or structure. | Includes compliments, expressions of satisfaction, or acknowledgments of the program's helpfulness. | Excludes neutral or negative comments, or feedback requesting changes. | "The program is good and very beneficial if truly implemented. According to me, filling out this form and applying it in daily life as per the module can have a positive impact." | Participant expresses positive feelings about the effectiveness of the mindfulness exercises in managing stress. |
