# Supplementary material for: Developing a mindfulness program for pre-clinical medical students in Indonesia: a mixed-methods study on suitability and appropriateness
Source: BMC Med Educ. 2025 Jul 17;25:1072. doi: 10.1186/s12909-025-07642-5 (PMC12272978; doi:10.1186/s12909-025-07642-5)
Supplement: Supplementary file 3 — Codebook [file 12909_2025_7642_MOESM3_ESM.docx]

**MINDFULNESS PROGRAM FOR PRE-CLINICAL MEDICAL STUDENTS IN INDONESIA (MPPMS-I)**

**CURRICULUM AND TEACHING GUIDE**

Developed by:

Denish Gunawan, MD


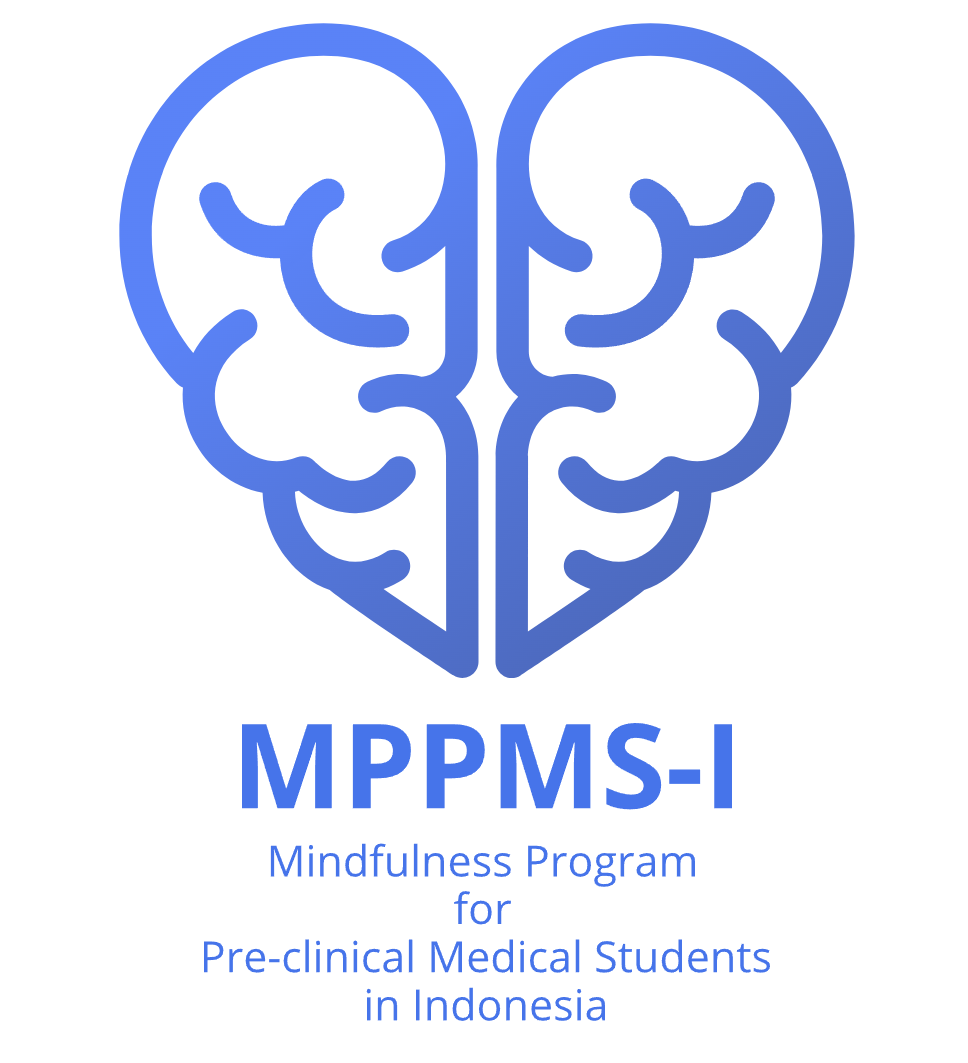


**Acknowledgments**

I would like to express my gratitude and acknowledgment to the pre-clinical medical students who provided valuable input in the development of this curriculum. I would also like to thank my thesis advisor, Professor Judson Brewer, and my thesis reader, Professor Sun Shu Fang. Additionally, I am grateful to my colleague, Lia Antico, PhD, my advisor from Atma Jaya Catholic University of Indonesia’s Faculty of Medicine and Health Sciences, dr. Nicholas Hardi, Sp. KJ, and my trainers during my qualification to teach the Mindfulness-based Stress Reduction program. Last but not least, I am thankful to Jon Kabat-Zinn, the founder of the Mindfulness-based Stress Reduction program. All of them have been an inspiration and generously shared their learnings and experiences, helping me develop this curriculum and teaching guide.

**Table of Contents**

Acknowledgements i

Table of Contents ii

Introduction iii

Aims iv

Summary of The Program 5

Session Description 9

Orientation session 9

Session One – Mind-Body Connection 12

Session Two – Perception and Responding to Stress 14

Session Three – How to Change Unhealthy Habits and Develop Healthy Habits 16

Session Four – Perfectionism and Self-compassion 18

Session Five – Mindful Communication 20

Session Six – Integrating Mindfulness into Daily Life 22

Session Materials 24

Orientation session 24

Session One – Mind-Body Connection 25

Session Two – Perception and Responding to Stress 30

Session Three – How to Change Unhealthy Habits and Develop Healthy Habits 33

Session Four – Perfectionism and Self-compassion 39

Session Five – Mindful Communication 41

Session Six – Integrating Mindfulness into Daily Life 44

Appendices 45

9 Dots Exercise 45

The Habit Mapper from Dr. Jud., Sharecare Inc. 46

Practice Notes 49

After Practice Journaling 49

Pleasant Experience Calendar 50

Unpleasant Experience Calendar 51

Difficult Communication Calendar 52

Reference 53

**Introduction**

Medical education is a demanding and stressful environment characterized by rigorous academic standards, heavy workloads, and psychological pressure. Unfortunately, these factors can negatively impact the physical and mental well-being of medical students, putting them at a higher risk for psychological distress, depression, burnout, anxiety, and suicidality compared to the general population (Anuradha et al., 2017; Arvant et al., 2021; Fahmi et al., 2018; Gupta et al., 2015; Perry et al., 2023; Polle & Gair, 2021; Shapiro et al., 2019). Globally, the prevalence rates of depression, anxiety, and burnout among medical students were estimated to be 27%, 34%, and 33.4-55% respectively (Nair et al., 2023; Wang et al., 2024). According to a cross-sectional study conducted in 2021 across 49 medical faculties in Indonesia, the prevalence of depression and anxiety were 16.8% and 43.7% respectively (Primatanti et al., 2023). Additionally, another cross-sectional study from 27 medical faculties in 2021 found that 35.5% of medical students experienced burnout (Cipta et al., 2022). Despite the numbers given, the rates of depression and anxiety in Asia countries are underreported compared to global standards due to the high stigma around mental health (Cuttilan et al., 2016)

Several systematic reviews and meta-analyses have demonstrated that Mindfulness-based Interventions (MBI) can produce small-to-moderate effects in reducing stress and psychological distress symptoms in medical students (Da Silva et al., 2023; Fahmi et al., 2018; Polle & Gair, 2021; Sekhar et al., 2021; Sperling et al., 2023). However, in Indonesia, there are only a few studies on the use of MBI among university students. These studies include the effect of mindfulness breathing for university students, the development of a culturally adapted internet-delivered mindfulness intervention for university students, and a pilot study to test the feasibility of a culturally adapted internet-delivered mindfulness program for university students (Komariah et al., 2022; Listiyandini et al., 2022, 2023). Nevertheless, to the best of the author's knowledge, no tailored mindfulness-based program has been developed specifically for medical students in Indonesia.

The author is proposing the development of a customized mindfulness-based mental health program for pre-clinical medical students at Atma Jaya Catholic University of Indonesia’s Faculty of Medicine and Health Sciences in Jakarta, Indonesia. The program, called "Mindfulness Program for Pre-clinical Medical Students in Indonesia (MPPMS-I)," is based on the framework of the Mindfulness-Based Stress Reduction (MBSR) program by Jon Kabat-Zinn, combined with other mindfulness-based programs such as the Unwinding Anxiety program by Judson Brewer and the Mindfulness Self-Compassion program by Christopher K. Germer and Kristin Neff. This program aims to provide pre-clinical medical students with mindfulness skills to handle various sources of stress in medical education and enhance their resilience to stress.

**Aims**

Several aims of this program are:

- - Understanding basic evidence of mindfulness
  - Knowing and experiencing various meditation techniques
  - Developing practical skills in mindfulness meditation to be incorporated into daily life
  - Learning how to cultivate positive emotions (compassion, forgiveness, kindness)
  - Understanding stress and reactivity towards stress
  - Managing stress and anxiety
  - Enhancing self-care behaviors
  - Developing effective work-life balance
  - Dealing with the needs to be perfect
  - Developing effective clinical interaction
  - Developing skills to deal with patients suffering

**Summary of The Program**

| Session | Pain points | Solutions | Experiential practice | Didactic materials | Life story |
| --- | --- | --- | --- | --- | --- |
| Orientation | Lack of understanding of mindfulness | Understanding the concept of mindfulness | Sitting meditation | Definition of mindfulness, what it is – and what it isn’t  Research related to mindfulness | None |
| 1  Mind-Body Connection | Not knowing oneself  Disconnection between mind and body | Understanding how mindfulness can increase awareness that leads to self-discovery  Understanding the interplay between mind and body influencing each other | Body scan  Sitting meditation | How mindfulness works and the core components of mindfulness  Triangle of Awareness  Zones of Experience  Stress: definitions & types of stressors  Autonomic Nervous Systems (ANS)  Stress reactions: Activation of the Sympathetic-Adreno-Medullar (SAM) axis and the Hypothalamic-Pituitary-Adrenal (HPA) axis | “I often don't realize the emotions or feelings I'm experiencing. For instance, when I'm stressed, I don't recognize that I'm stressed. However, my work becomes unfinished, my performance declines and my emotions become unstable. I feel like I'm being chased by something, but I don't know what it is. I don't know why I'm stressed or that what I'm feeling is stress. I want to better understand myself and my emotions.” – P033 |
| 2  Perception and Responding to Stress | Lack of awareness of how perception influences stress levels  Stressors and challenges in medical school, such as homework, reading materials, and exams that disrupt work-life balance  Habitual reactions to stress | Understanding how perception influences stress levels  Differentiating between reacting to stress and responding to it mindfully | 9 dots exercise  Walking meditation  S.T.O.P. meditation  Sitting meditation | Perception and optical illusions  Discussion of 9 dots solution and its meaning  Stress reaction vs. Stress response | “When I faced the OSCE exam in the 6th semester of medical school, I suddenly panicked and became stressed before entering the exam room. As a result, in the first station of the exam, I forgot some steps in performing catheter insertion, spoke haltingly, and my hands trembled, causing delays in unwrapping the equipment. After finishing the first station, I realized that I had practiced multiple times and was competent in performing the procedure, but the stress and panic caused me to make many mistakes. I then calmed myself down and took a few deep breaths before entering the next exam room, successfully completing the subsequent stations.” – P044 |
| 3  How to Change Unhealthy Habits and Develop Healthy Habits | Unhealthy habits such as poor diet, lack of exercise, and inadequate sleep that affect well-being | Understanding habit loop  Understanding the three gears to break free from unhealthy habit loops  Understanding the importance of healthy habits | Yoga  R.A.I.N. meditation  Sitting meditation | Habit loop model  Three gears to break free from unhealthy habit loops  The importance of a healthy diet  The importance of exercising  The importance of sleep | “This semester, I neglected my physical condition. I focused too much on academics, neglecting my body’s needs. Even though my weight didn’t significantly increase due to reduced eating frequency, I often studied late into the night and only slept 2-3 hours a day. This resulted in sleeping during lectures and a cycle of extended study periods at home, which further decreased my sleep. After the exams, I realized the negative impact on my body from these bad habits.” – P040 |
| 4  Perfectionism and Self-compassion | Stress from perfectionism which is related to performance anxiety, self-criticism, guilt, and shame  Empathy fatigue | Understanding how perfectionism is related to stress and anxiety  Self-compassion as the antidote to perfectionism | Self-compassion meditation  Sitting meditation | Perfectionism trait and its relationship with stress and anxiety  Self-compassion definition and its components  Compassion vs. Empathy | “At the start of my studies, I was often dissatisfied with the assignments I completed because I felt they were not good enough. This led me to spend excessive time on a single task, causing stress due to the piling work and the need to do everything perfectly. Eventually, I realized that while tasks need to be done well, they should not be viewed as burdens, so I could complete everything efficiently and enjoy my academic experience more.” – P040 |
| 5  Mindful Communication | Poor communication leads to misunderstanding and strained relationship | Mindful communication | Loving-kindness meditation  Sitting meditation | Communication styles  Three foundations of mindful communication  The Nonviolent Communication | “I once joined a committee where frequent disputes occurred among team members. Initially, as the chairperson, I would often side with what I thought was right. But after self-reflection, I realized I needed to listen to others' opinions first, even if it was unpleasant, and still show respect.” – P034 |
| 6  Integrating Mindfulness into Daily Life | Difficulty in continuing mindfulness practices amidst the busy medical school life | Guidance on mindfulness practice continuation | Reflection on practice by journaling  Choiceless awareness meditation  Sitting meditation | Guidance on how to continue the practice | None |

**Session Description**

**Orientation Session**

The orientation session serves as an introduction to the program.

During the orientation, a brief sitting meditation with options for foci of attention will be introduced.

After the orientation session, the teacher will have a brief meeting with each student to go over the orientation questionnaire paperwork (refer to the appendices) and address any questions and/or concerns about the program. This also provides an opportunity for the teacher to connect with the students directly and understand their interests and level of commitment. If there are signs that a student might not be suitable for the program, the teacher should be prepared to provide a referral to a therapist.

**Orientation Session Teaching Plan**

| Time | Activity |
| --- | --- |
| 0’-20’ | Introduction to Mindfulness   - Definition of mindfulness - What mindfulness is and what mindfulness isn’t - Research related to mindfulness |
| 20-35’ | Logistics   - Dates and times - Community guidelines:  1. Confidentiality: Everything shared in the class stays in the class. 2. No taking pictures or recording without permission 3. Being kind and respectful towards the teacher and other students (watching diversity, equity, and inclusivity) 4. Refraining from giving advice 5. Sharing only from personal experience. Using the “I” statement. 6. If you happen to hurt someone, accept and acknowledge the person’s experience 7. Creating space for vulnerability  - Self-care:  1. Wearing comfortable clothes 2. Changing positions to manage pain or discomfort, or even stopping the exercise if the experience of strong emotions or sensations is overwhelming  - Home practice: Committing to doing home practice by finding time to practice and being willing to meet oneself despite personal resistance, fatigue, boredom, restlessness, fear, pain, and whatever arises. Remembering about self-care. - Risks and Benefits  1. Risks:   The practice can incite or exacerbate strong physical, emotional, psychological, or relational issues.   - Physical risks: Students who have physical conditions, including musculoskeletal conditions, prior injuries or surgeries, or conditions that impact balance or movement should be encouraged to check with their healthcare provider for adapting any of the yoga postures. - Emotional/psychological risks: During practice, experiences that have carried strong emotions or have had a strong psychological impact may surface. Any history of recent loss, life change, or trauma might make emotions more acute and labile. - Social risks: Session or practice may impact family time and school life. Setting time to practice may shift some responsibilities. Those around you may be surprised or uncomfortable with new behavior and/or attitudes.   If any of the risks emerge, students are encouraged to notify the teacher privately   1. Benefits:  - Improved sense of well-being – even if symptoms or conditions stay the same - Symptoms reduction - Increased focus and reduced distraction - Emotional balance - Increased joy and contentment - Increased resilience - Increased appreciation and awareness of inner and outer resources |
| 35’-45’ | Sitting meditation with options for anchors (e.g., breath, body position, or sound) |
| 45’-60’ | Large group discussion |
| 60’-65’ | Closing  Brief sitting meditation with options for anchors (e.g., breath, body position, or sound) |

**Session One – Mind-Body Connection**

The first session of the 6-week program focuses on the theme of the mind-body connection.

The session starts by welcoming all students to the 6-week program and inviting them to appreciate the time and effort they are dedicating to self-discovery. This session aims to help students rediscover the connection between the mind and the body through mindfulness. The theme emphasizes that the mind and body are not separate entities; they can influence each other. During this session, students will learn about mindfulness through three main mindfulness components: attention control, emotion regulation, and self-awareness (Tang et al., 2015). Additionally, students will be introduced to zones of experience, stress concepts, and stress reactions.

In the first session, we will introduce the body scan practice. This practice aims to deepen the mind-body connection by helping students become more aware of bodily sensations and emotions. Many people are so disconnected from their bodies that they can't detect subtle sensations and the emotions that may accompany them.

**Session One Teaching Plan**

| Time | Activity |
| --- | --- |
| 0’-10’ | Checking in and reviewing the definition of mindfulness |
| 10’-25’ | Body scan practice |
| 25’40’ | Discussion |
| 40’-60’ | Didactic:   - How mindfulness works, core components of mindfulness: attention control, emotion regulation, and self-awareness - Triangle of Awareness (i.e., body sensations, emotions, and thoughts) - Zones of Experience (i.e, Comfort zone, challenge zone, and overwhelm zone) - Stress: definitions & types of stressors - Autonomic Nervous Systems (ANS) - Stress reactions: Activation of the Sympathetic-Adreno-Medullar (SAM) axis and the and Hypothalamic-Pituitary-Adrenal (HPA) axis |
| 60’-65’ | Life story related to mind-body connection:  “I often don't realize the emotions or feelings I'm experiencing. For instance, when I'm stressed, I don't recognize that I'm stressed. However, my work becomes unfinished, my performance declines, and my emotions become unstable. I feel like I'm being chased by something, but I don't know what it is. I don't know why I'm stressed or that what I'm feeling is stress. I want to better understand myself and my emotions.” – P033 |
| 65’-70’ | Review of home practice   - Practicing daily 15-minute of body scan practice alternated with sitting meditation for 6 days - Eating mindfully one time - Working on 9 dots exercise *(see appendix: 9 Dots Practice)* - Journaling after the practice *(see appendix: Practice Notes)* |
| 70’-75’ | Checking-out  Brief sitting meditation with options for anchors (e.g., breath, body position, or sound) |

**Session Two – Perception and Responding to Stress**

The second session focuses on the theme of perception and how to respond to stress. Perception is related to how we view things, including how students perceive stressors and challenges in medical school, such as homework, reading materials, and exams, as well as their inner resources and capacities. These stressors and challenges can disrupt students' work-life balance.

In this session, students will learn how their skewed perception contributes to stress and may be the root cause of their stress. The session aims to teach students how mindfulness can change their perception of stressors and help them learn to respond to stress rather than habitually reacting to it.

In this session, we introduce walking meditation as another form of meditation to demonstrate that mindfulness doesn't have to be stationary all the time. Additionally, we will also introduce STOP meditation (Stop, Take a breath, Observe, Proceed) as a simple and quick tool for students to use whenever they encounter stressors.

**Session Two Teaching Plan**

| Time | Activity |
| --- | --- |
| 0’-10’ | Checking in and discussing the homework of the past week |
| 10’-25’ | Walking meditation |
| 25’-40’ | Discussion |
| 40’-60’ | Didactic:   - Perception and optical illusions - Discussion of 9 dots solution and its meaning - Stress reaction vs. Stress response - Introducing S.T.O.P. (Stop, Take a breath, Observe, Proceed) meditation |
| 60’-65’ | Life story related to perception and responding to stress:  “When I faced the OSCE exam in the 6th semester of medical school, I suddenly panicked and became stressed before entering the exam room. As a result, in the first station of the exam, I forgot some steps in performing catheter insertion, spoke haltingly, and my hands trembled, causing delays in unwrapping the equipment. After finishing the first station, I realized that I had practiced multiple times and was competent in performing the procedure, but the stress and panic caused me to make many mistakes. I then calmed myself down and took a few deep breaths before entering the next exam room, successfully completing the subsequent stations.” – P044 |
| 65’-70’ | Review of home practice   - Practicing daily 15 minutes of body scan alternated with sitting meditation/walking meditation practice for 6 days - Filling out the pleasant events calendar *(see appendix: Practice Notes)* - Practicing S.T.O.P. meditation when you meet stressful events - Journaling after the practice *(see appendix: Practice Notes)* |
| 70’-75’ | Checking-out  Brief sitting meditation with options for anchors (e.g., breath, body position, or sound) |

**Session Three – How to Change Unhealthy Habits and Develop Healthy Habits**

The third session covers the topic of developing healthy habits. Due to the demanding nature of medical school and the accompanying stress, students often do not pay attention to their eating patterns and choices; fail to exercise; and do not get sufficient sleeping time and quality. Over time, these habits can have a negative impact on both their physical and mental health, becoming an additional source of stress and creating a harmful cycle. Breaking this cycle requires awareness.

In this session, students will learn about the habit loop model (trigger, behaviors, and results) adapted from Judson Brewer’s book “Unwinding Anxiety” and the three gears to break free from the habit loop of unhealthy habits (mapping the elements of the habit loop; updating the brain’s reward value; choosing a bigger better offer). The goal of this material is to encourage students to recognize and be aware of their unhealthy habits and use mindfulness to develop healthier habits as part of self-care.

In this session, we will introduce yoga meditation as a way to be present with the body through movements, which has a different quality from meditation in a still position. Additionally, we will introduce RAIN (Recognize, Allow, Investigate, Note) meditation as another simple tool to practice. This meditation can help you stay present whenever stress or unhealthy habit loops arise.

**Session Three Teaching Plan**

| Time | Activity |
| --- | --- |
| 0’-10’ | Checking in and discussing the homework of the past week |
| 10’-25’ | Standing yoga meditation |
| 25’-40’ | Discussion |
| 40’-60’ | Didactic:   - Habit loop model (trigger, behaviors, and results) - Three gears to break free from unhealthy habit loops (mapping the elements of the habit loop; knowing the results of the behavior; choosing a bigger better offer) - The importance of a healthy diet - The importance of exercising - The importance of sleep - R.A.I.N. (Recognize, Allow, Investigate, Note) meditation |
| 60’-65’ | Life story related to developing healthy habits:  “This semester, I neglected my physical condition. I focused too much on academics, neglecting my body’s needs. Even though my weight didn’t significantly increase due to reduced eating frequency, I often studied late into the night and only slept 2-3 hours a day. This resulted in sleeping during lectures and a cycle of extended study periods at home, which further decreased my sleep. After the exams, I realized the negative impact on my body from these bad habits.” – P040 |
| 65’-70’ | Review of home practice   - Practicing daily 15 minutes of body scan alternated with sitting meditation/walking meditation/standing or lying down yoga practice for 6 days - Filling out the unpleasant events calendar *(see appendix: Practice Notes)* - Practicing R.A.I.N. meditation when you meet stressful events - Practicing the Habit Loop Mapper *(see appendix: The Habit Mapper by Dr. Jud’s Sharecare Inc.)* - Journaling after the practice *(see appendix: Practice Notes)* |
| 70’-75’ | Checking-out  Brief sitting meditation with options for anchors (e.g., breath, body position, or sound) |

**Week Four – Perfectionism and Self-compassion**

In the fourth session, we will be discussing the topic of perfectionism and self-compassion. Medical students often have a perfectionist trait, which involves having high personal standards and expectations, as well as being overly self-critical or self-judgmental. This trait can manifest as anxiety about their performance in medical school, being hard on themselves when they fall short of their expectations, and feeling guilt and shame for making mistakes. While this trait may seem beneficial for their success, it actually creates a lot of stress.

In this session, we will explore the concept of self-compassion as an antidote to perfectionism. The aim of introducing this topic is to raise awareness among students to acknowledge, allow, and/or accept things as they are as well as to remind students to show themselves the kindness they deserve.

In this session, self-compassion meditation is introduced to cultivate compassion towards oneself, similar to offering compassion to others when witnessing their suffering.

**Week Four Teaching Plan**

| Time | Activity |
| --- | --- |
| 0’-10’ | Checking in and discussing the homework of the past week |
| 10’-25’ | Self-compassion meditation |
| 25’-45’ | Discussion |
| 45’-60’ | Didactic:   - Perfectionism trait and its relationship with stress and anxiety - Self-compassion definition and its components (i.e., self-kindness, common humanity, and mindfulness) - Compassion vs. Empathy |
| 60’-65’ | Life story related to perfectionism and self-compassion:  “At the start of my studies, I was often dissatisfied with the assignments I completed because I felt they were not good enough. This led me to spend excessive time on a single task, causing stress due to the piling work and the need to do everything perfectly. Eventually, I realized that while tasks need to be done well, they should not be viewed as burdens, so I could complete everything efficiently and enjoy my academic experience more.” – P042 |
| 65’-70’ | Review of home practice   - Practicing daily 15 minutes of body scan alternated with sitting meditation/walking meditation/standing or lying down yoga practice/self-compassion meditation for 6 days - Becoming aware of automatic habitual stress reactions during the week - Filling out the difficult communications calendar *(see appendix: Practice Notes)* - Journaling after the practice *(see appendix: Practice Notes)* |
| 70’-75’ | Checking-out  Brief sitting meditation with options for anchors (e.g., breath, body position, or sound) |

**Session Five – Mindful Communication**

The fifth session focuses on the importance of incorporating mindfulness into communication. Developing strong communication skills is crucial for medical students, as many interpersonal issues and medical errors stem from misunderstandings. These misunderstandings can strain relationships with colleagues, seniors, teachers, and patients, adding to the existing stressors of medical school. By improving communication skills, students can build better relationships with others.

During this session, students will learn how mindfulness can enhance their interactions with others. They will be encouraged to integrate mindfulness into their speaking and listening habits, while also exploring any automatic reactive patterns that may hinder effective communication.

In this session, we introduce loving-kindness meditation to cultivate the natural capacity for kindness towards oneself and others.

**Session Five Teaching Plan**

| Time | Activity |
| --- | --- |
| 0’-10’ | Checking in and discussing the homework of the past week |
| 10’-25’ | Loving-kindness meditation |
| 25’-45’ | Discussion |
| 45’-60’ | Didactic:   - Communication styles (i.e., passive, aggressive, passive-aggressive, and assertive communications) - Three foundations of mindful communication (i.e., presence, intention, attention) - The Nonviolent Communication (i.e., Observations, Feelings, Needs, Requests) |
| 60’-65’ | Life story related to mindful communication:  “I once joined a committee where frequent disputes occurred among team members. Initially, as the chairperson, I would often side with what I thought was right. But after self-reflection, I realized I needed to listen to others' opinions first, even if it was unpleasant, and still show respect.” – P034 |
| 65’-70’ | Review of home practice   - Practicing daily 15 minutes of body scan alternated with sitting meditation/walking meditation/standing or lying down yoga practice/self-compassion meditation/lovingkindness meditation for 6 days - Becoming aware of moments in difficult communications - "Be mindful of what you consume: 1. Physically, such as food or drink; or 2. Through your senses, such as media, TV, internet, etc. Pay attention to the impact of what you take in and evaluate the result. Is it the outcome you desire? Put into practice what you've learned and observe the effects of one of these "inputs." - Journaling after the practice *(see appendix: Practice Notes)* |
| 70’-75’ | Checking-out  Brief sitting meditation with options for anchors (e.g., breath, body position, or sound) |

**Session Six – Integrating Mindfulness into Daily Life**

The sixth session is the final session of this program. This session will discuss the topic of integrating mindfulness into daily life after practicing for the past 6 weeks. Students are encouraged to continue practicing mindfulness amid their busy medical school program in order to reap the most benefits. The last session is also meant to appreciate the students’ discipline, commitment, and energy they have displayed during the program.

During this session, students will be asked to reflect on their practice and to share their experiences in the large group over the past 6 weeks. Additionally, students will be given guidance on how they can continue and make the practice their own.

In this session, choiceless awareness meditation is introduced. In choiceless awareness practice, the present moment becomes the primary object of attention instead of focusing on a specific object. This practice allows the cultivation of awareness of the ever-changing state of the present moment by noticing whatever arises in the body and mind, whether sensations, sounds, thoughts, or emotions.

**Session Six Teaching Plan**

| Time | Activity |
| --- | --- |
| 0’-10’ | Checking in and discussing the homework of the past week |
| 10’-25’ | Choiceless awareness meditation |
| 25’-30’ | Journaling for self-reflection on mindfulness practice and the events of the past 6 weeks. |
| 30’-55’ | Discussion |
| 55’-65’ | Didactic:   - Guidance on how to continue the practice |
| 65’-70’ | Final thoughts |
| 70’-75’ | Checking-out  Brief sitting meditation with options for anchors (e.g., breath, body position, or sound) |

**Session Materials**

**Orientation Session**

- **Definition of mindfulness**

Mindfulness: “The awareness that arises through paying attention on purpose, in the present moment, and nonjudgmentally to the unfolding of experience moment by moment.” – Jon Kabat-Zinn

- **What mindfulness is and what mindfulness isn’t (Kabat-Zinn, 2013)**


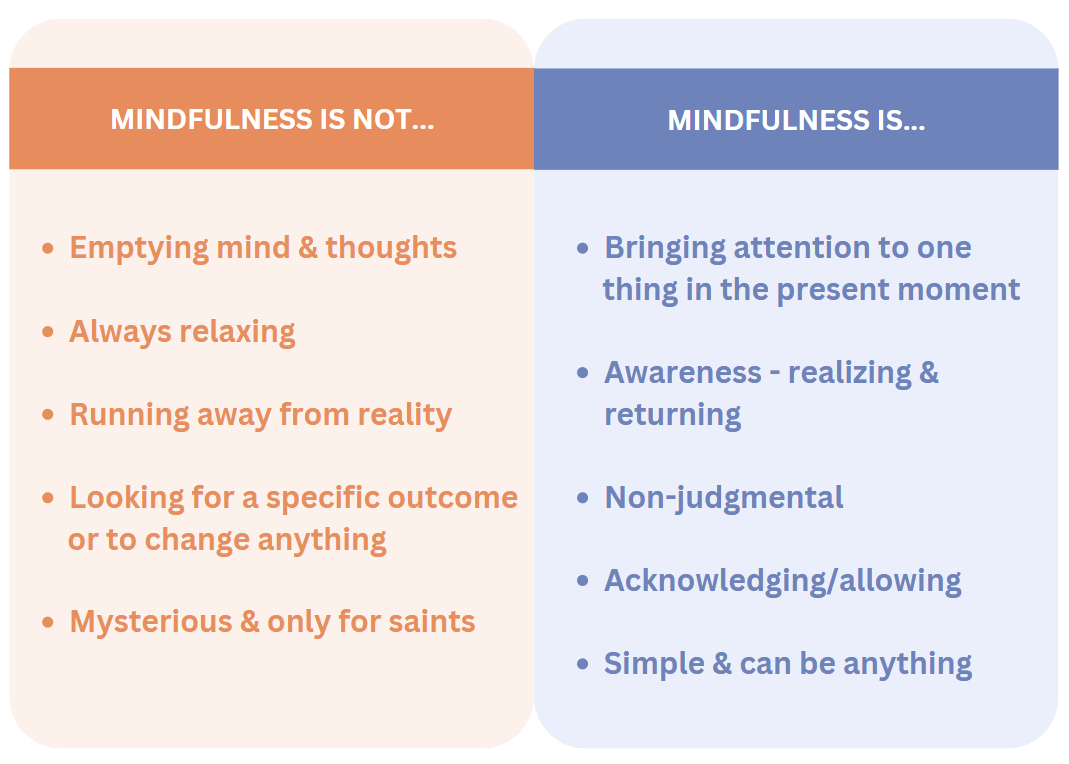


Figure 1. What mindfulness is and what mindfulness isn’t (Kabat-Zinn, 2013)

- **Research related to mindfulness**

Benefits of mindfulness:

- - Reduces stress, anxiety, and depression symptoms (Ding et al., 2023; Khoury et al., 2015)
  - Improves chronic pain (Hilton et al., 2017)
  - Improves sleep quality (Chen et al., 2020)
  - Reduces high blood pressure (Lee et al., 2020)
  - Improves working memory, executive function, and cognitive flexibility (Whitfield et al., 2022)
  - Helps with weight reduction (Carrière et al., 2018)
  - Enhances immune system (Black & Slavich, 2016)

**Session One – Mind-Body Connection**

- **How mindfulness works (Tang et al., 2015)**

Mindfulness meditation enhances self-regulation through three main components: enhanced attention control, improved emotion regulation, and altered self-awareness (diminished self-referential processing and enhanced body awareness) (Tang et al., 2015).


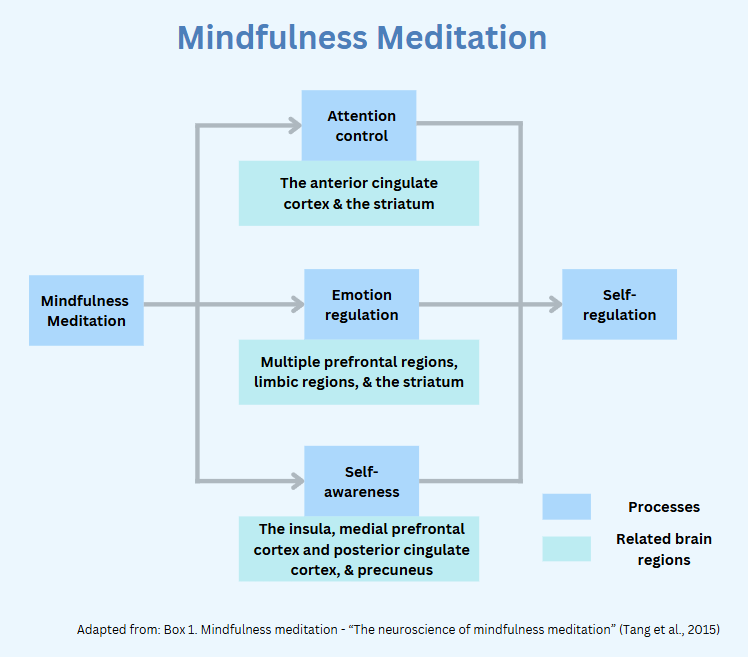


Figure 2. Mindfulness Meditation

Adapted from: Box 1. Mindfulness meditation (Tang et al., 2015).

- **Triangle of Awareness (Loucks, 2022)**


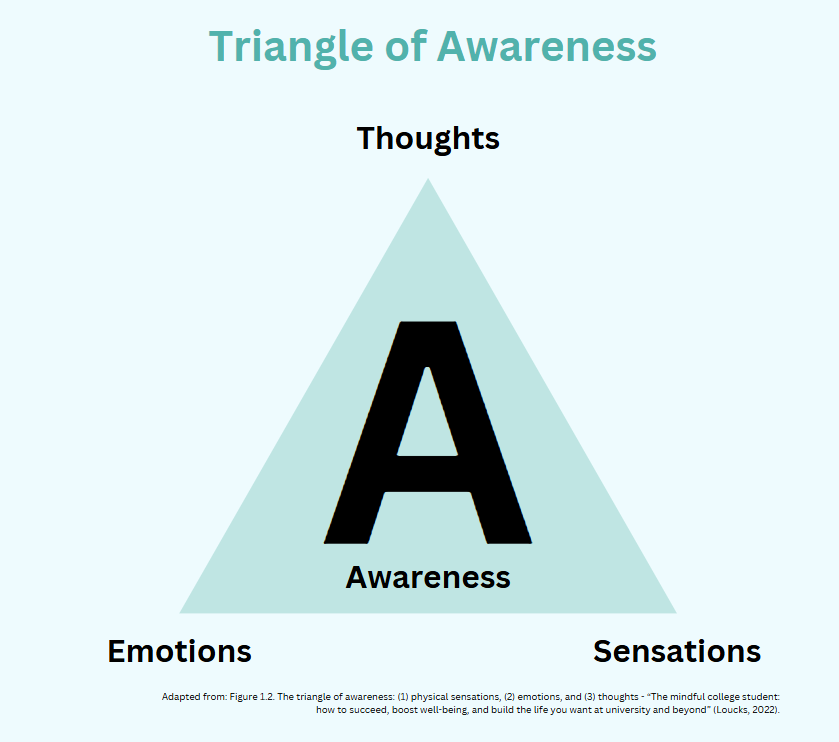


Figure 3. Triangle of Awareness

Adapted from: Figure 1.2. The triangle of awareness: (1) physical sensations, (2) emotions, and (3) thoughts (Loucks, 2022).

- **Zones of Experience**


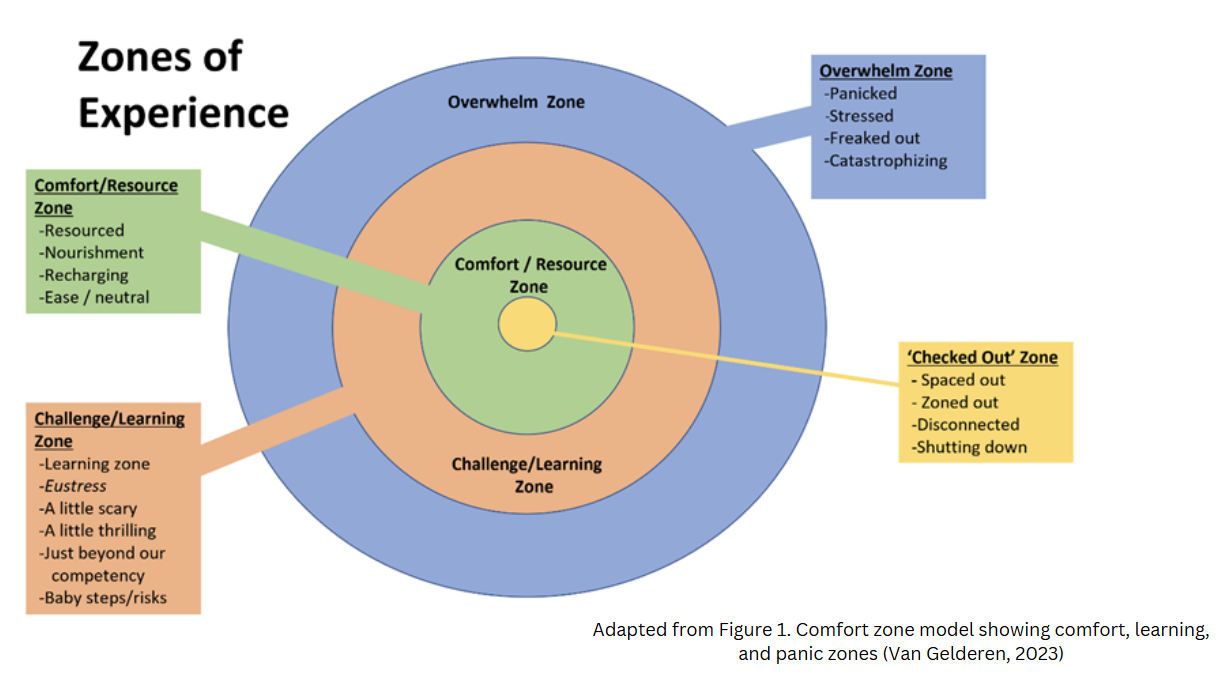


Figure 4. Zones of Experience

Adapted from Figure 1. Comfort zone model showing comfort, learning, and panic zones (Van Gelderen, 2023).

- Stress

1. Definitions
   - “The non-specific response of the body to any demand placed upon it.” – Hans Selye (Kabat-Zinn, 2013)
   - Stress occurs when a person perceives the demands of environmental stimuli to be greater than their ability to meet, mitigate, or alter those demands. (Lazarus et al., 1985)
2. Type of stressors by Hans Selye: Eustress vs. Distress (Kabat-Zinn, 2013; Manosso et al., 2022)
   - Eustress (helpful stress): manifested as feelings of excitement, fulfillment, meaning, satisfaction, and well-being; a perception of a stressor as positive and challenging
   - Distress (unhelpful stress): manifested as a sense of suffering and decreased quality of life


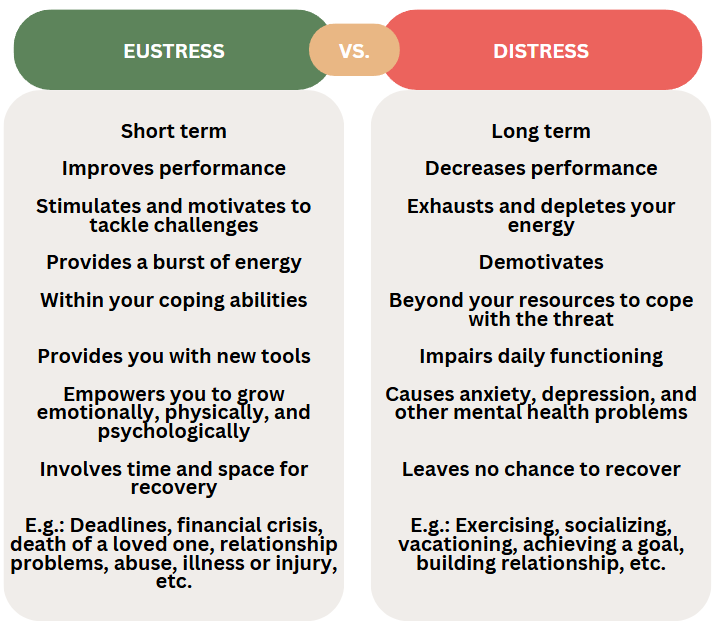


Figure 5. Type of stressors by Hans Selye: Eustress vs. Distress (Kabat-Zinn, 2013; Manosso et al., 2022)

**“It is not the potential stressor itself but how you perceive it and then how you handle it that will determine whether or not it will lead to stress.” – Dr. Seligman (Kabat-Zinn, 2013)**

- **Autonomic Nervous System**


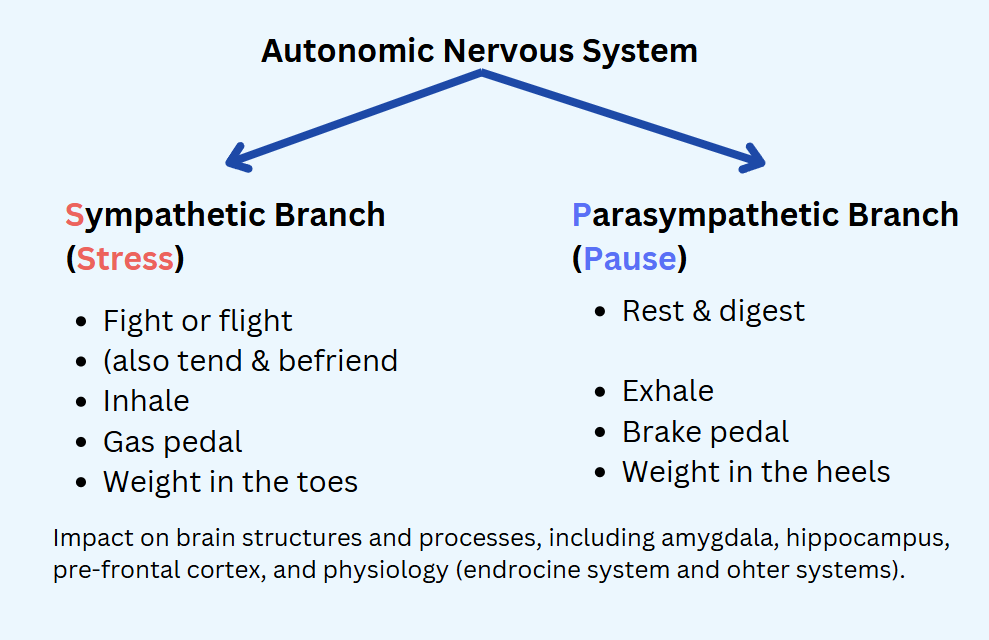


Figure 7. Autonomic Nervous System (LeBouef et al., 2024)

- **Stress Reactions: Activation of the Sympathetic-Adreno-Medullar (SAM) axis and the and Hypothalamic-Pituitary-Adrenal (HPA) axis (Ulrich-Lai & Herman, 2009)**


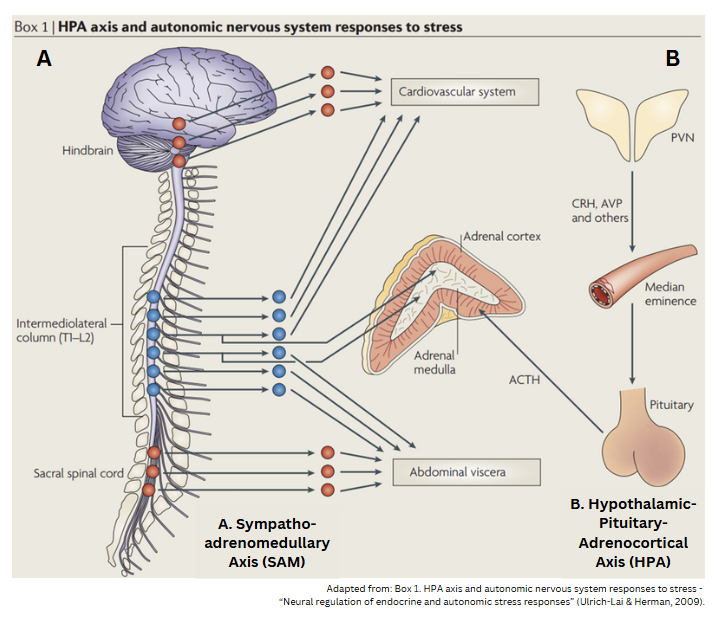


Figure 8. HPA axis and Autonomic Nervous System Responses to Stress

Adapted from: Box 1. HPA axis and autonomic nervous system responses to stress - Neural regulation of endocrine and autonomic stress responses (Ulrich-Lai & Herman, 2009).

- **The Stress System (Godoy et al., 2018)**


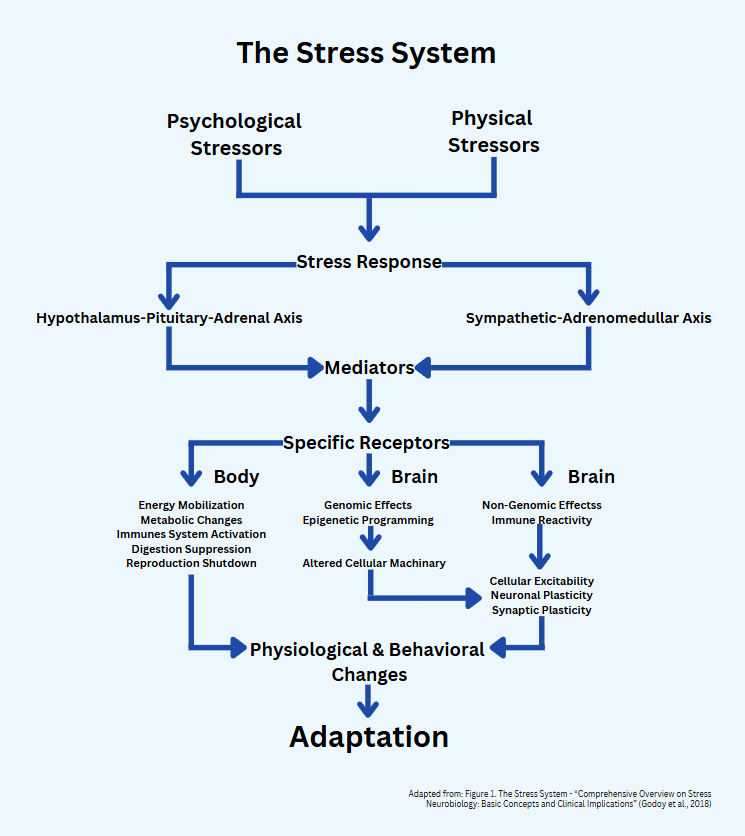


Figure 9. The Stress System

Adapted from: Figure 1. The Stress System - A Comprehensive Overview on Stress Neurobiology: Basic Concepts and Clinical Implications (Godoy et al., 2018).

**Session Two – Perception and Responding to Stress**

- **Perception and Optical Illusions**

Definition: Perception is the process by which the brain interprets and organizes sensory information from the environment to produce a meaningful experience of the world – Berkeley Well-being Institute. (*Perception*, n.d.)


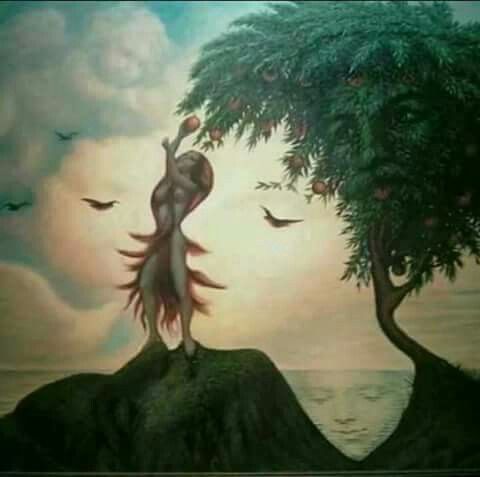


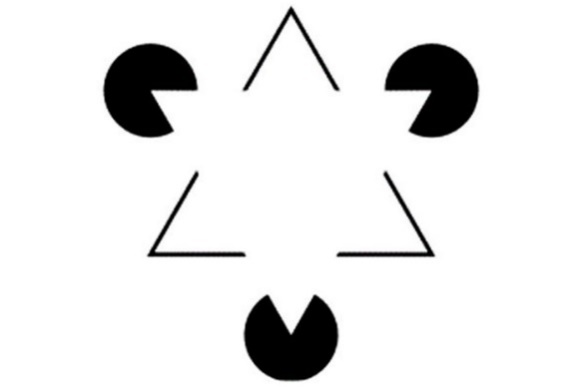


How many triangles are in this picture?

Figure 10. Optical Illusion (1)

What do you see in this picture?

Source of images:

<https://openclipart.org/detail/217727/triangle-optical-illusion>

Figure 11. Optical Illusion (2)

https://www.pinterest.com/pin/462604192975681076/

- **9 dots exercise (Kabat-Zinn, 2013)**

Draw 4 straight lines that connect

all 9 dots without

lifting your pen at all

**
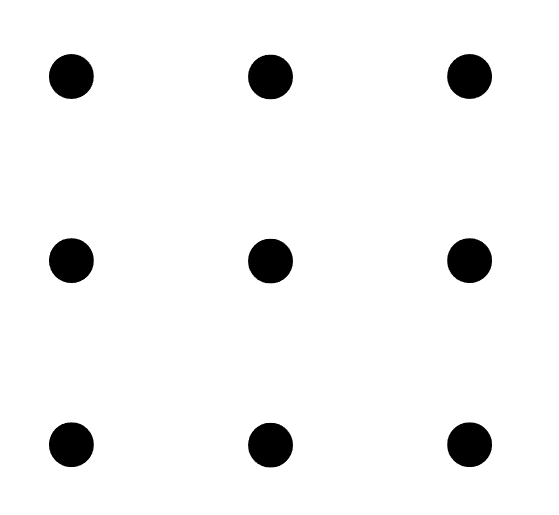
**

Figure 12. 9 dots (Kabat-Zinn, 2013)

- **Stress Reaction vs. Stress Response (Kabat-Zinn, 2013)**


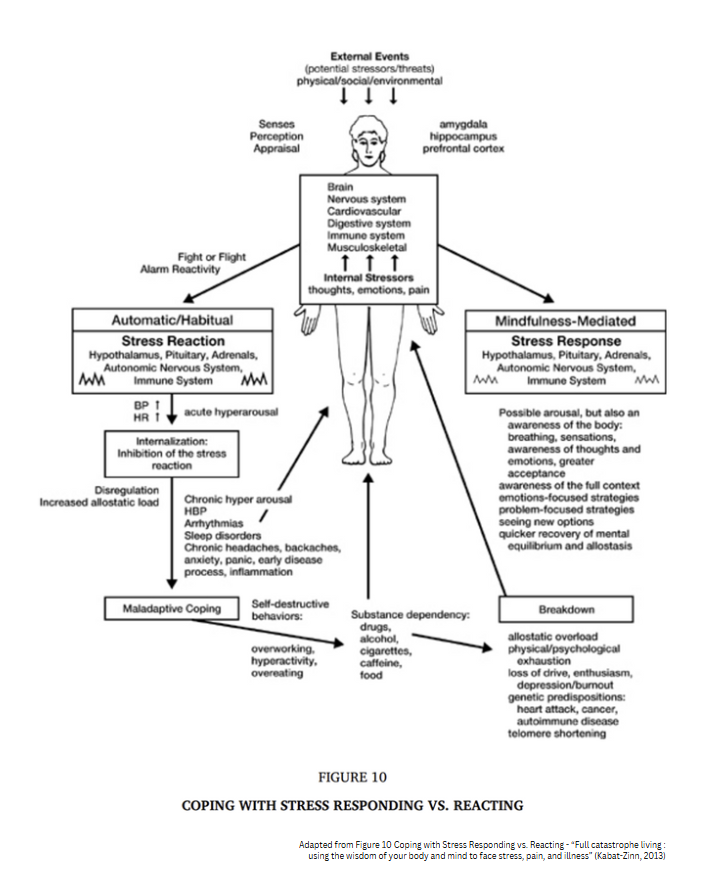


Figure 13. Coping with Stress Responding vs. Reacting

Adapted from: Figure 10 Coping with Stress Responding vs. Reacting (Kabat-Zinn, 2013).

- **S.T.O.P. Meditation**  **(*Practice*, n.d.)**


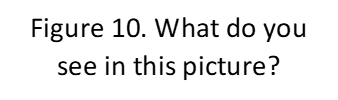

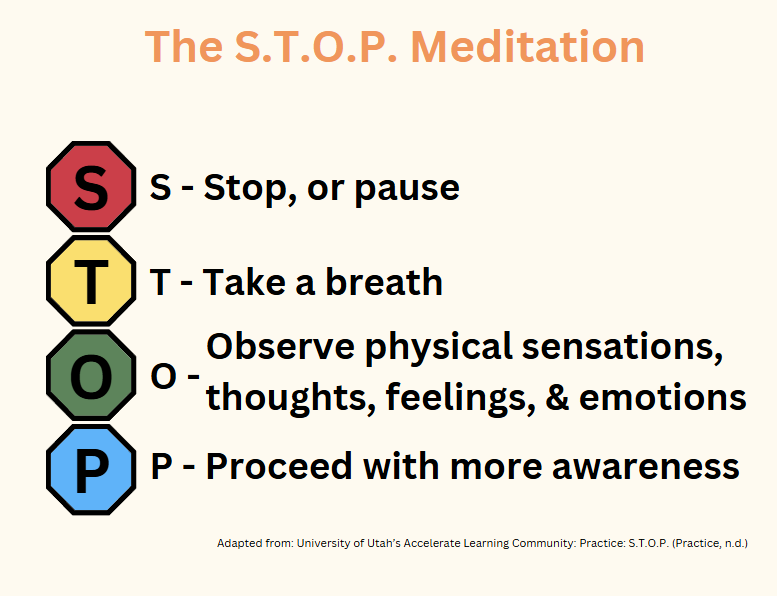


Figure 14. S.T.O.P. Meditation (*Practice*, n.d.)

S.T.O.P. meditation is an informal mindfulness practice that allows us to take a breath and check in to see how we’re doing. It can assist us in shifting from states of distraction and automatic pilot to being present and intentional (*Practice*, n.d.).

**Session Three – How to Change Unhealthy Habits and Develop Healthy Habits**

- **Habit Loop Model (Brewer, 2021)**

The habit loop is a concept from the field of behavioral science that describes the cycle through which habits form and persist. It consists of three key elements:

1. Trigger (Cue): This is what starts the habit. It could be a thought, an emotion, a physical sensation, or something you see or encounter. For instance:

- Feeling stressed out after a long day.
- Seeing a package of cookies on the counter.
- Not having healthy food in the house.

1. Behavior (Routine): The actual habit itself. It can be a physical action (like eating cookies or chips) or a mental behavior (such as worrying or self-judgment).
2. Result (Reward): How you feel after the behavior. In the short term, it might feel good (e.g., the taste of cookies). However, in the long term, it may not be as beneficial (e.g., contributing to unhealthy weight gain).

**
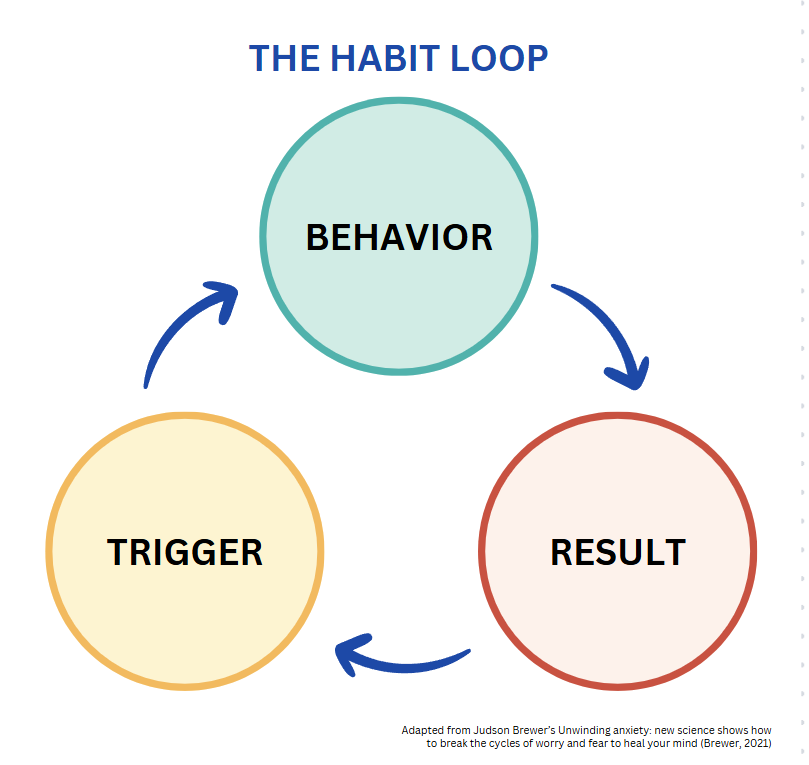
**

Figure 15. The Habit Loop

Adapted from: Unwinding anxiety: new science shows how to break the cycles of worry and fear to heal your mind (Brewer, 2021).

- **Three gears to break free from unhealthy habit loops (Brewer, 2021)**

1. First gear: Mapping your habit loop and recognizing its three components clearly: trigger, behavior, and reward
2. Second gear: Updating your brain’s reward value
3. Third gear: Finding that Bigger, Better Offer (BBO) for your brain


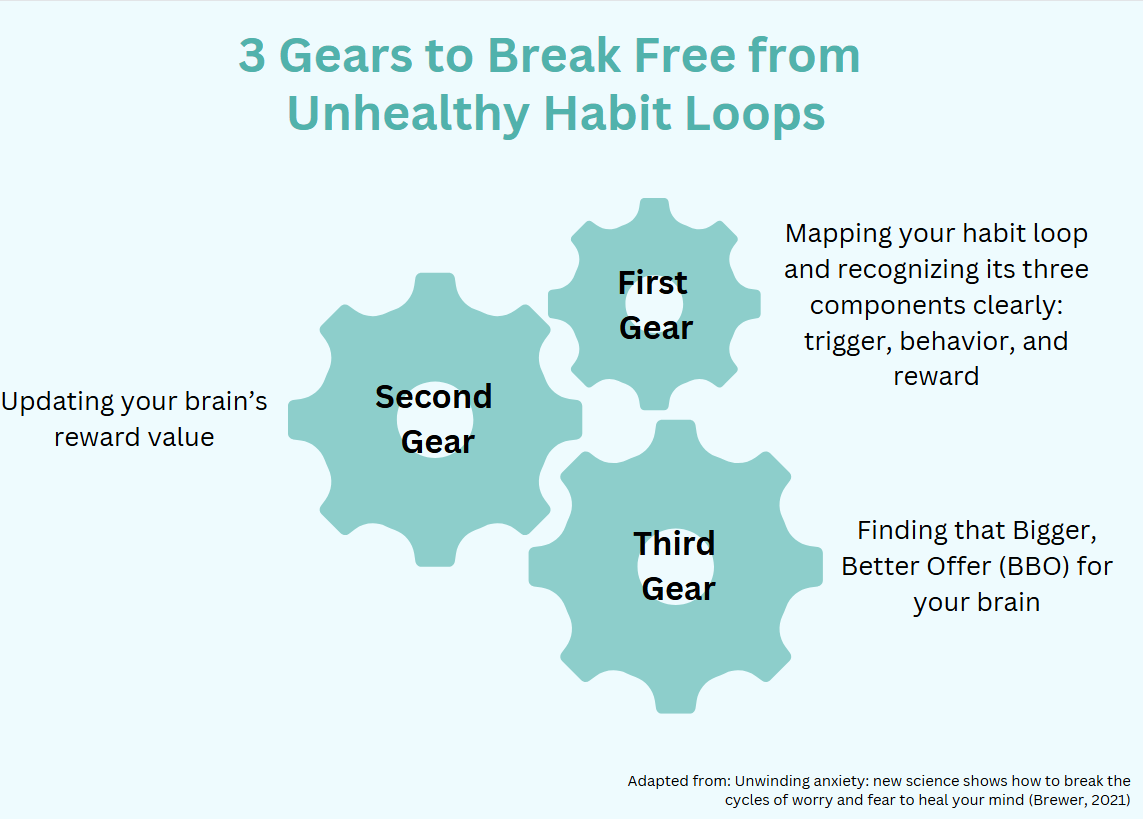


Figure 16. 3 Gears to Break Free from Unhealthy Habit Loops

Adapted from: Unwinding anxiety: new science shows how to break the cycles of worry and fear to heal your mind (Brewer, 2021).

- **The importance of a healthy diet**

1. Benefits (CDC, 2024; *Healthy Diet*, 2020):

- Reduces the risk of non-communicable diseases, such as heart disease, type 2 diabetes, and some cancers;
- Supports muscles and strengthens bones;
- Keeps skin, teeth, and eyes healthy;
- Boosts immunity;
- Supports healthy pregnancies and breastfeeding;
- Helps the digestive system function; and
- Helps achieve and maintain a healthy weight.

1. Recommendations from WHO (*Healthy Diet*, 2020):

- To meet the needs of energy, protein, vitamins, and minerals through a varied diet, largely plant-based, and balancing energy intake with expenditure;
- Obtaining the largest amount of energy from carbohydrates, mainly through legumes and wholegrain cereals;
- Reducing total fats to less than 30% of total energy intake, shifting fat intake away from saturated and trans fat to unsaturated fats, and eliminating industrial trans fats from the diet;
- Reducing free sugars to less than 10% (ideally 5%) of total energy intake;
- Limiting sodium intake to less than 2 grams per day (equivalent to 5 grams of salt); and
- Consuming at least 400 grams of vegetables and fruit per day in adults and children above 10, and 250–350 grams per day in younger children.
- **The importance of exercising**

1. Benefits (CDC, 2024; *Physical Activity*, 2022.; *Real-Life Benefits of Exercise and Physical Activity*, 2020):

- Improves muscular and cardiorespiratory fitness;
- Improves bone and functional health;
- Reduces the risk of non-communicable diseases, such as hypertension, coronary heart disease, stroke, diabetes, various types of cancer (including breast cancer and colon cancer), and depression;
- Reduces the risk of infectious diseases, such as flu, pneumonia, and COVID-19;
- Helps maintain a healthy body weight;
- Strengthen your bones and muscles;
- Improves sleep;
- Reduces levels of stress and anxiety;
- Improves mood and overall emotional well-being;
- Helps control blood sugar;
- Helps support daily living activities and independence for people with disabilities;
- Reduces the risk of falls as well as hip or vertebral fractures; and
- Increases the chances of living longer.

1. Recommendations from WHO for adults aged 18-64 years(*Physical Activity*, 2022.):

- At least 150-300 minutes of moderate-intensity, or 75-150 minutes of vigorous-intensity aerobic physical activity weekly, or an equivalent combination;
- Muscle-strengthening activities should be done on two or more days a week; and
- Should limit the amount of time spent being sedentary.
- **The importance of sleep**

1. Benefits (*8 Health Benefits of Sleep*, 2022; *How Sleep Clears the Brain*, 2015):

- Improves mental function, such as learning and memory consolidation;
- Improves mood and emotional regulation;
- Reduces stress, anxiety, and depression;
- Helps energy conservation, growth, and healing; and
- Strengthens immunity; and
- Maintains cardiovascular health and regulates blood sugar

1. Recommendation from the National Sleep Foundation (thensf, 2020):

Adults: Between the ages of 18 and 64, adults should aim for seven to nine hours of nightly sleep

1. Sleep hygiene *(next page)*

**Sleep Hygiene**

**What is Sleep Hygiene?** Sleep hygiene is the term used to describe good sleep habits. Considerable research has gone into developing a set of guidelines and tips designed to enhance good sleeping, and there is much evidence to suggest that these strategies can provide long-term solutions to sleep difficulties.

1.
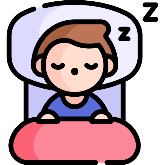
**Get regular.** Go to bed and get up at more or less the same time every day, even on weekends and days off!
2. **Get up & try again.** Go to sleep only when tired. If you haven’t been able to fall asleep after 30 minutes, get up and do something calming (not stimulating) until you feel sleepy, then return to bed and try again.
3.
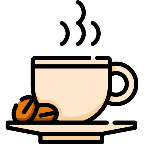
**Avoid caffeine & nicotine.** Avoid consuming any caffeine (coffee, tea, soda, chocolate) or nicotine (cigarettes) for at least 4-6 hours before going to bed. These act as stimulants and interfere with falling asleep
4. **Avoid alcohol.** Avoid alcohol for at least 4-6 hours before bed because it interrupts the quality of sleep.
5. **Bed is for sleeping.** Do not use your bed for anything other than sleeping and sex, so that your body comes to associate bed with sleep.
6.
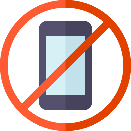
**Electronics Curfew.** Don’t use back-lit electronics 60 minutes prior to bed, as the artificial light prevents hormones and neurons that promote sleep.
7. **The right space.** Make your bed and bedroom quiet and comfortable for sleeping. An eye mask and earplugs may help block out light and noise.
8. **No naps.** Avoid taking naps during the day. If you can’t make it through the day without a nap, make sure it is for less than an hour and before 3 pm.
9.
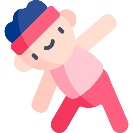
**Sleep rituals.** Develop rituals to remind your body that it is time to sleep, like relaxing stretches or breathing exercises for 15 minutes before bed.
10. **No clock-watching.** Checking the clock during the night can wake you up and reinforce negative thoughts such as “Oh no, look how late it is, I’ll never get to sleep.”
11. **Keep the daytime routine the same.** Even if you have a bad night's sleep it is important that you try to keep your daytime activities the same as you had planned. That is, don’t avoid activities because you feel tired. This can reinforce insomnia.

Adapted from Western Australia Department of Health’s Center Centre for Clinical Interventions: Sleep Hygiene (*Sleep & Insomnia Self-Help Resources - Information Sheets*, n.d.)

Source of images:

www.flaticon.com/free-icons/sleep" title="sleep icons”

www.flaticon.com/free-icons/food" title="food icons”

www.flaticon.com/free-icons/no-phone"

www.flaticon.com/free-icons/workout" title="

- **R.A.I.N. Meditation (Brewer, 2021)**


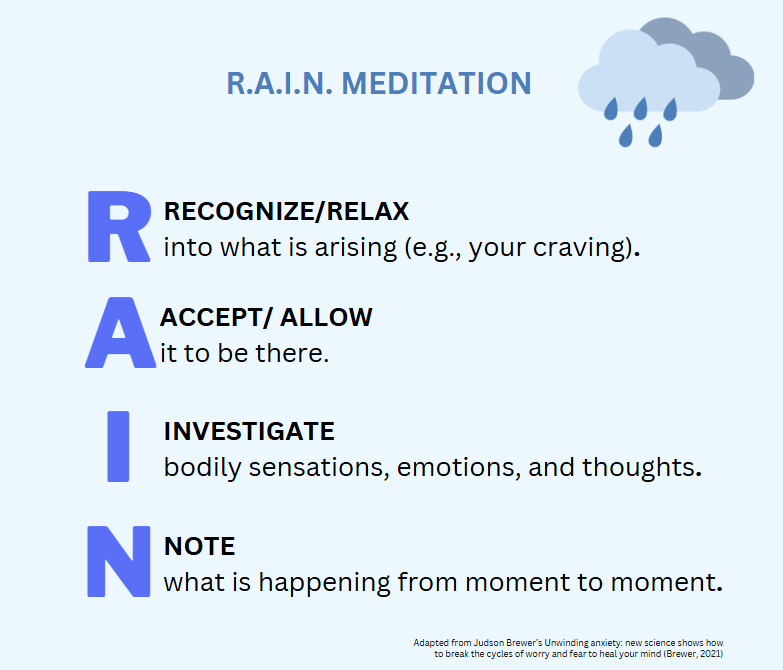


Figure 17. R.A.I.N. Meditation

Adapted from: Unwinding anxiety: new science shows how to break the cycles of worry and fear to heal your mind (Brewer, 2021).

**Session Four – Perfectionism and Self-compassion**

- **Perfectionism trait and its relationship with stress and anxiety**
  1. Definition: Perfectionism is defined as a combination of excessively high personal standards and overly critical self-evaluations. (Curran & Hill, 2019)
  2. 3 types of perfectionism based on the direction of perfectionistic beliefs and behaviors (Curran & Hill, 2019):
- Self-oriented: individuals attach irrational importance to being perfect, hold unrealistic expectations of themselves, and are punitive in their self-evaluations.
- Socially prescribed: individuals believe their social context is excessively demanding, that others judge them harshly, and that they must display perfection to secure approval.
- Other-oriented: individuals impose unrealistic standards on those around them and evaluate others critically.

1. Perfectionism is correlated with anxiety, obsessive-compulsive disorder (OCD), and depression (Lunn et al., 2023).

- **Self-compassion definition and its components**
  1. Definition: Self-compassion is being “kind and understanding when confronted with personal failings”. It means treating yourself with the same empathy and support that you would extend to a close friend during difficult times: noticing the suffering; empathizing or suffering with yourself; offering kindness and understanding. (“What Is Self-Compassion?,” n.d.)
  2. 3 components of self-compassion (Finlay-Jones et al., 2023):
- Self-kindness vs. Self-judgment: Treating yourself with care and understanding rather than harsh self-criticism.
- Common humanity vs. isolation: Recognizing that suffering and personal failures are part of the shared human experience.
- Mindfulness vs. Overidentification: Maintaining a balanced awareness of your suffering without suppressing or exaggerating it.


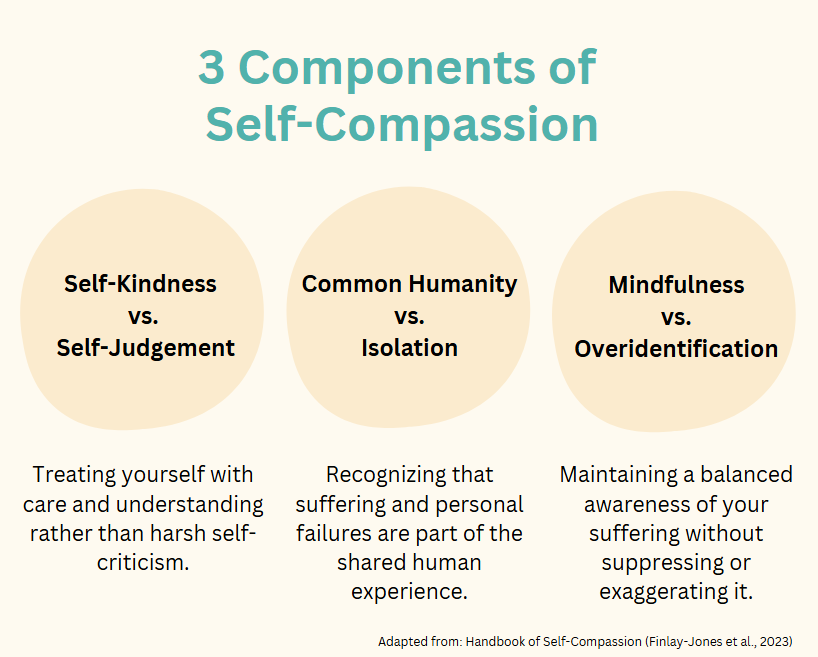


Figure 18. 3 Components of Self-Compassion

Adapted from: Handbook of Self-Compassion (Finlay-Jones et al., 2023).

- **Compassion vs. Empathy (*Compassion vs. Empathy*, n.d.)**


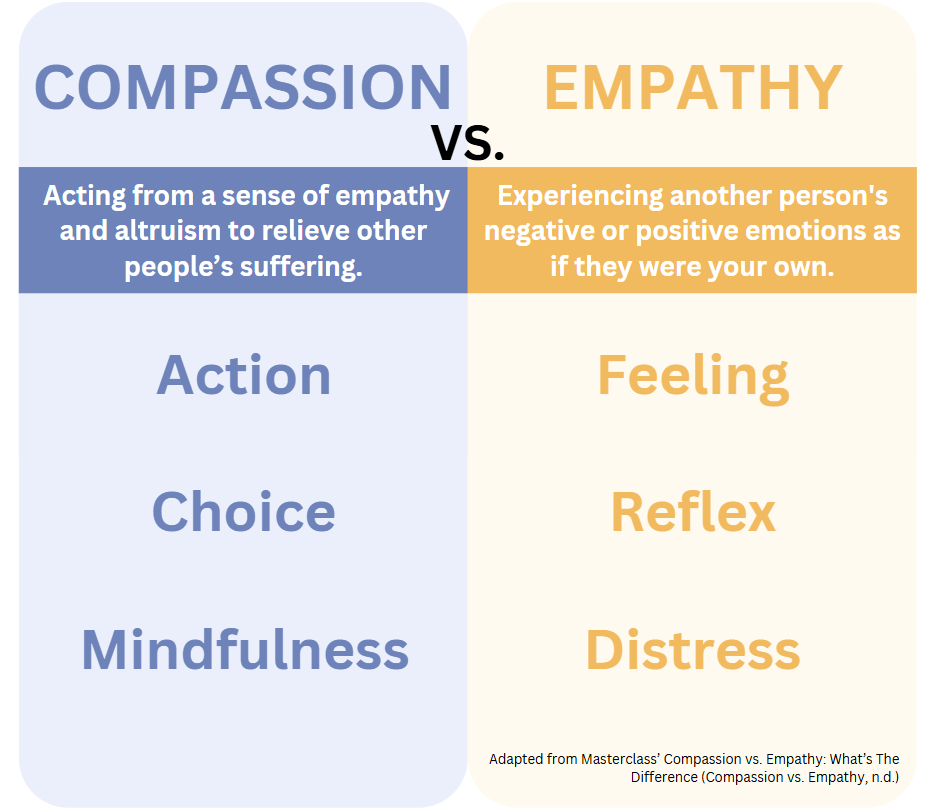


Figure 19. Compassion vs. Empathy

Adapted from: Masterclass’ Compassion vs. Empathy: What’s The Difference (*Compassion vs. Empathy*, n.d.).

**Session Five – Mindful Communication**

- **Communication styles (*Understanding Your Communication Style*, n.d.)**

1. Passive Communication
   - Not expressing your feelings or needs, neglecting your own rights, and allowing others to do the same
   - Letting others make decisions to avoid tension or conflict
   - Often results in misunderstandings, built-up anger, or resentment
   - Can be safer when a conflict might escalate to violence
   - Examples: saying “I’m okay with whatever you want to do”; body language: avoiding eye contact or looking down
2. Aggressive Communication

- Expressing feelings, needs, and ideas at others' expense; ignoring their rights to assert your own
- Defensive or hostile when confronted
- Often alienates and hurts others
- Can quickly meet your needs
- Examples: saying “this is what we’re doing” or “get over it”; body language: crossing arms, eye-rolling, or finger pointing

1. Passive-Aggressive Communication

- Appearing passive but subtly acting out anger
- Using sarcasm, indirect communication, or avoiding conversation to control others
- Showing little regard for others' rights, needs, or feelings
- Examples: passive statements and body language followed by the "silent treatment," spreading rumors, or sabotaging other’s efforts

1. Assertive Communication

- Direct and honest expression of thoughts and feelings
- Respecting others' feelings, ideas, and needs while asserting your own
- May be ineffective with individuals who threaten your safety
- Often misinterpreted as aggressive, particularly among Americans and women
- Examples: using “I” statements like “I feel...when you…and I need for you to...”; body language: maintaining eye contact, straight posture, and relaxed gestures


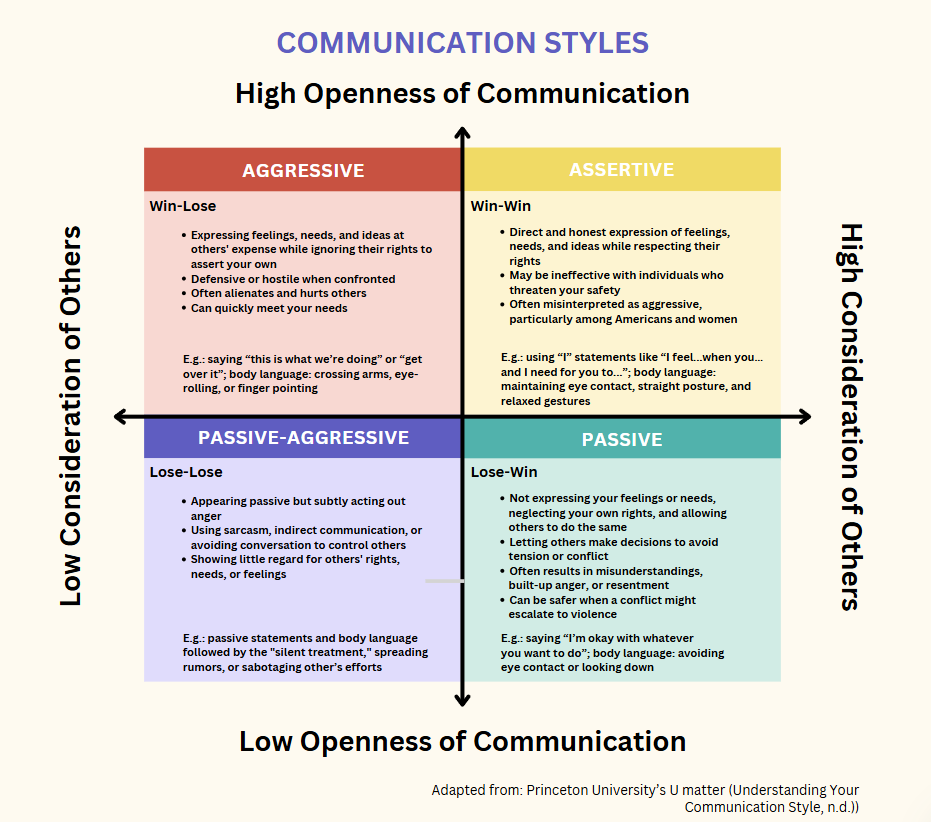


Figure 20. Communication Styles

Adapted from: Princeton University’s U matter (Understanding Your Communication Style, n.d.).

- **Three foundations of mindful communication (*How Does Mindfulness Improve Communication?*, 2022)**

1. Presence: grounding our awareness in the body

2. Intention: cultivating an orientation in heart with a purpose to understand

3. Attention: training to focus in specific areas of our experience to gain deeper perspective


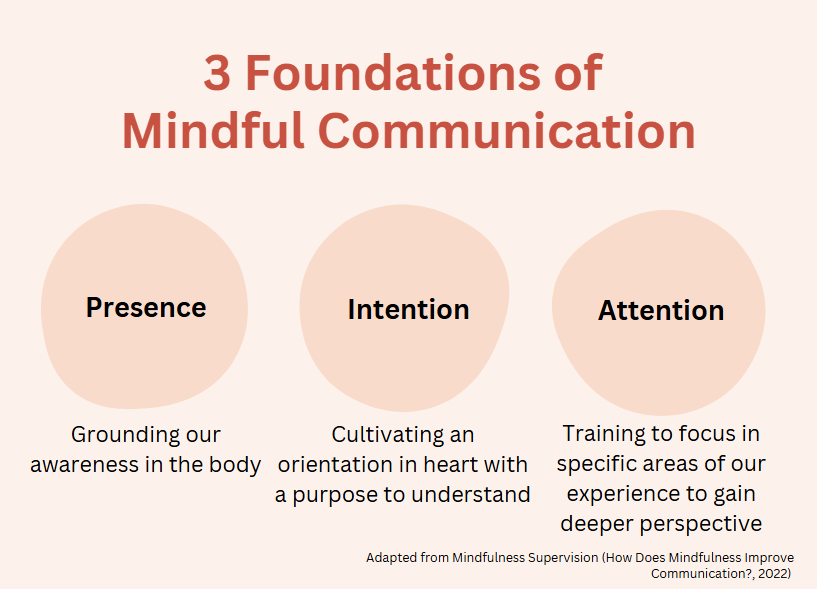


Figure 21. 3 Foundations of Mindful Communication

Adapted from: Mindfulness Supervision (*How Does Mindfulness Improve Communication?*, 2022).

- **4 Parts of Nonviolent Communication (*4-Part Nonviolent Communication (NVC) - PuddleDancer Press*, n.d.)**
  - 1. Observations: Begin by observing facts without judgment or exaggeration. Focus on concrete actions or events.
    2. Feelings: Identify and express your emotions related to those observations.
    3. Needs: Recognize the underlying needs, wants, and values that drive your feelings.
    4. Requests: Make explicit requests based on your needs, rather than making demands.

**
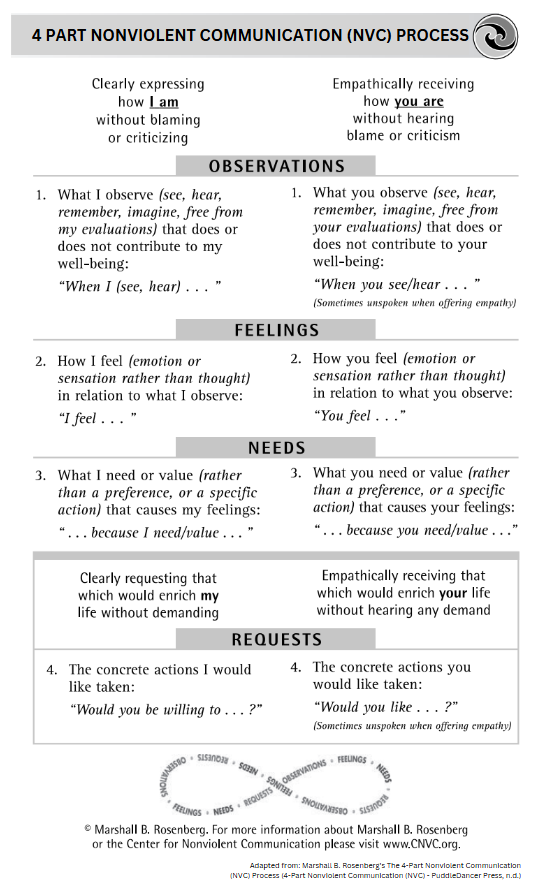
**

Figure 22. 4 Parts of Nonviolent Communication

Adapted from: Marshall B. Rosenberg’s The 4-Part Nonviolent Communication

(NVC) Process (*4-Part Nonviolent Communication (NVC) - PuddleDancer Press*, n.d.)

**Session Six – Integrating Mindfulness into Daily Life**

- **Guidance on how to continue the practice**

1. Finding time to consider and write down:
   - “What do I need to continue my mindfulness practice?”
   - “What will support my ongoing commitment to practice mindfulness?”
2. Emphasize practicality and simplicity, such as:
   - Buying a meditation cushion
   - Setting up space in one’s house to practice meditation or yoga
   - Downloading a timer app on one’s phone
   - Downloading meditation recordings on one’s phone
3. Finding someone or a community to share with about the practice and to help support accountability

**Appendices**

**9 Dots Exercise**

Draw 4 straight lines that connect all 9 dots without lifting your pen at all.

**
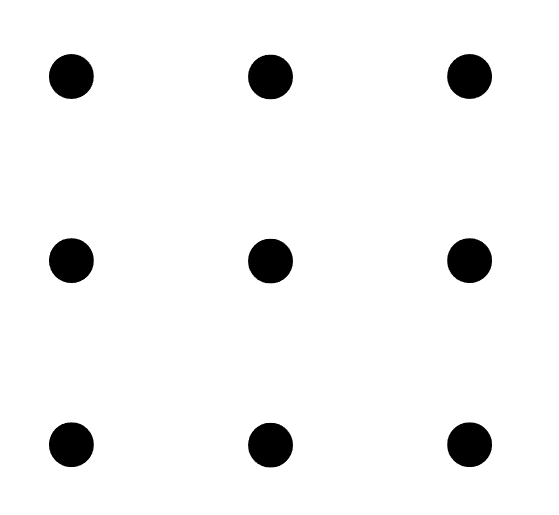
**

**The Habit Mapper by Dr. Jud’s Sharecare Inc.**

**Adapted from Dr. Jud’s Habit Mapper, Sharecare Inc. Unwinding Anxiety. (2021)**

To ***change habits*** you need to know ***how they work***.

The **Habit Mapper** is a simple tool that will help you understand **how your brain works**, so you can work with it to **change your behavior** and **break bad habits**.

**All habits have three elements:** a **TRIGGER**, a **BEHAVIOR**, and a **RESULT**.

**2**

The **RESULT** is how you feel

after the behavior. In the

short term, this might feel

good, but in the long term,

not as much.

The **TRIGGER** is what starts

the habit. It can be something

you see or a place you visit, or

just a thought, emotion, or

physical sensation.

**3**

**1**

The **BEHAVIOR i**s the habit

itself. It could be a physical

behavior like biting your nails

or too much time on social

media. It can also be a mental

behavior like worrying or

self-judgement.

**Example: Imagine you have a habit of eating too many cookies or potato chips:**

**RESULTS**

**In the moment:**

- Feel less hungry
- Salt/sugar makes you feel good

**Over time:**

- Feel less in control
- May contribute to unhealthy weight gain

**BEHAVIOR**

Eating a whole bag of chips or cookies.

**TRIGGERS**

**Emotions/Sensations:**

- Stressed out after a long day
- Feel restless or hungry after a meal

**Situations:**

- See a package on counter
- No healthy food in house

By breaking down your habit into its three parts, you can begin to **recognize how the habit starts**, and **how unrewarding** and **unhelpful it is for you**. This is **‘’new information’’** for your brain, and **is the most important step in breaking bad habits.**

**Let’s get started!**

**
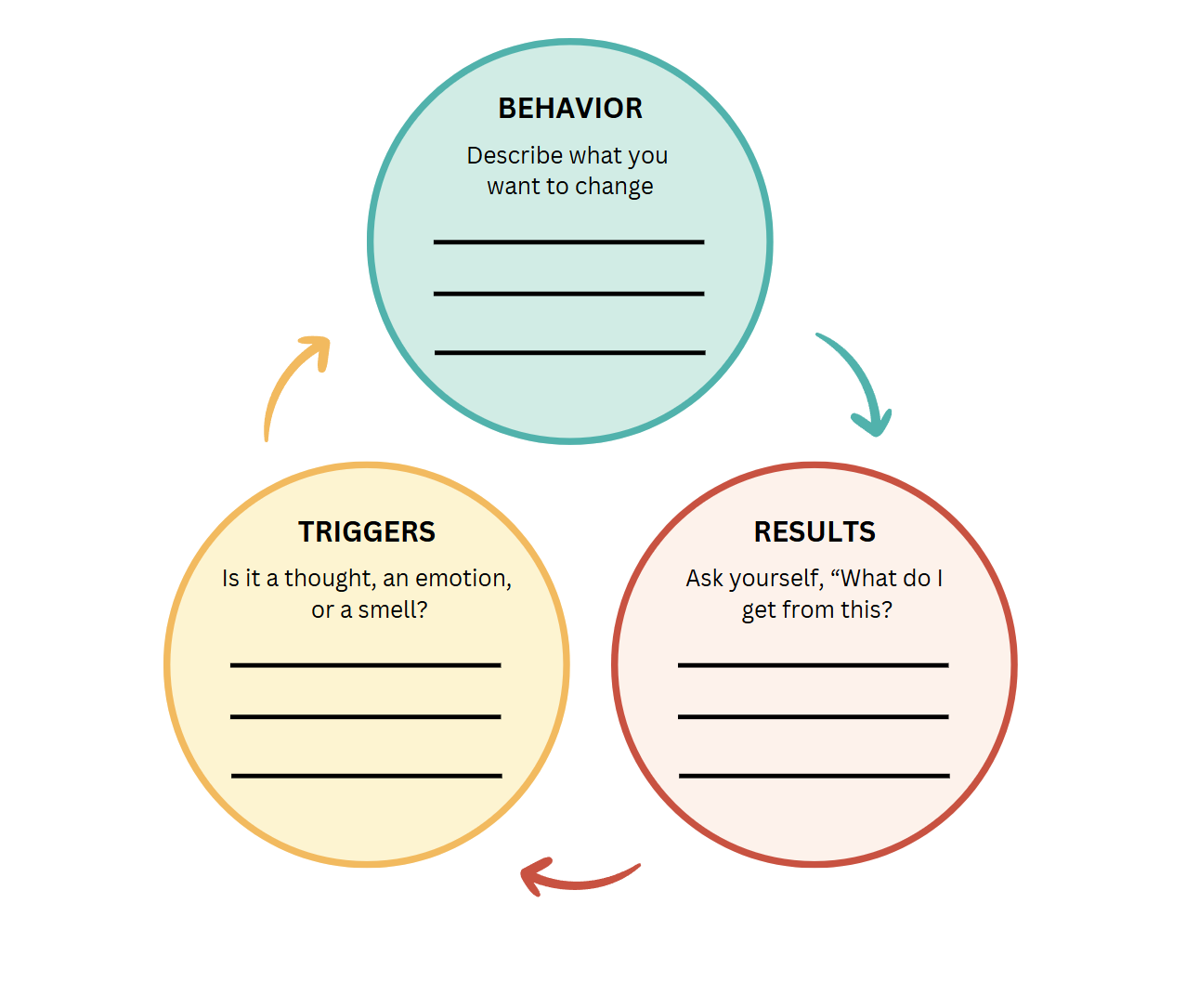
**

Now that you know how to map a habit by breaking

it down into its three parts, you can apply this

technique to other bad habits or unwanted

behaviors. Try it out below.

**
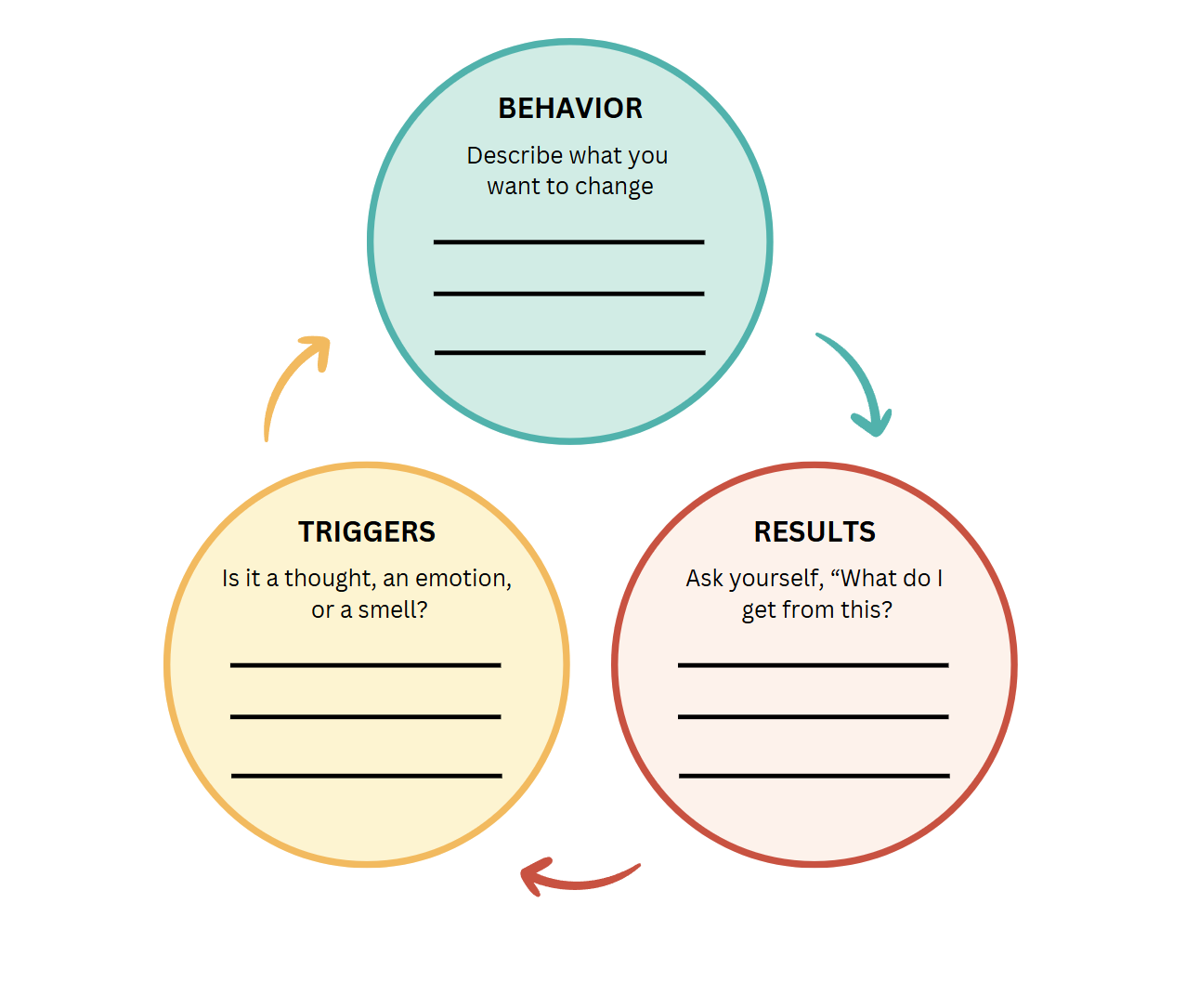

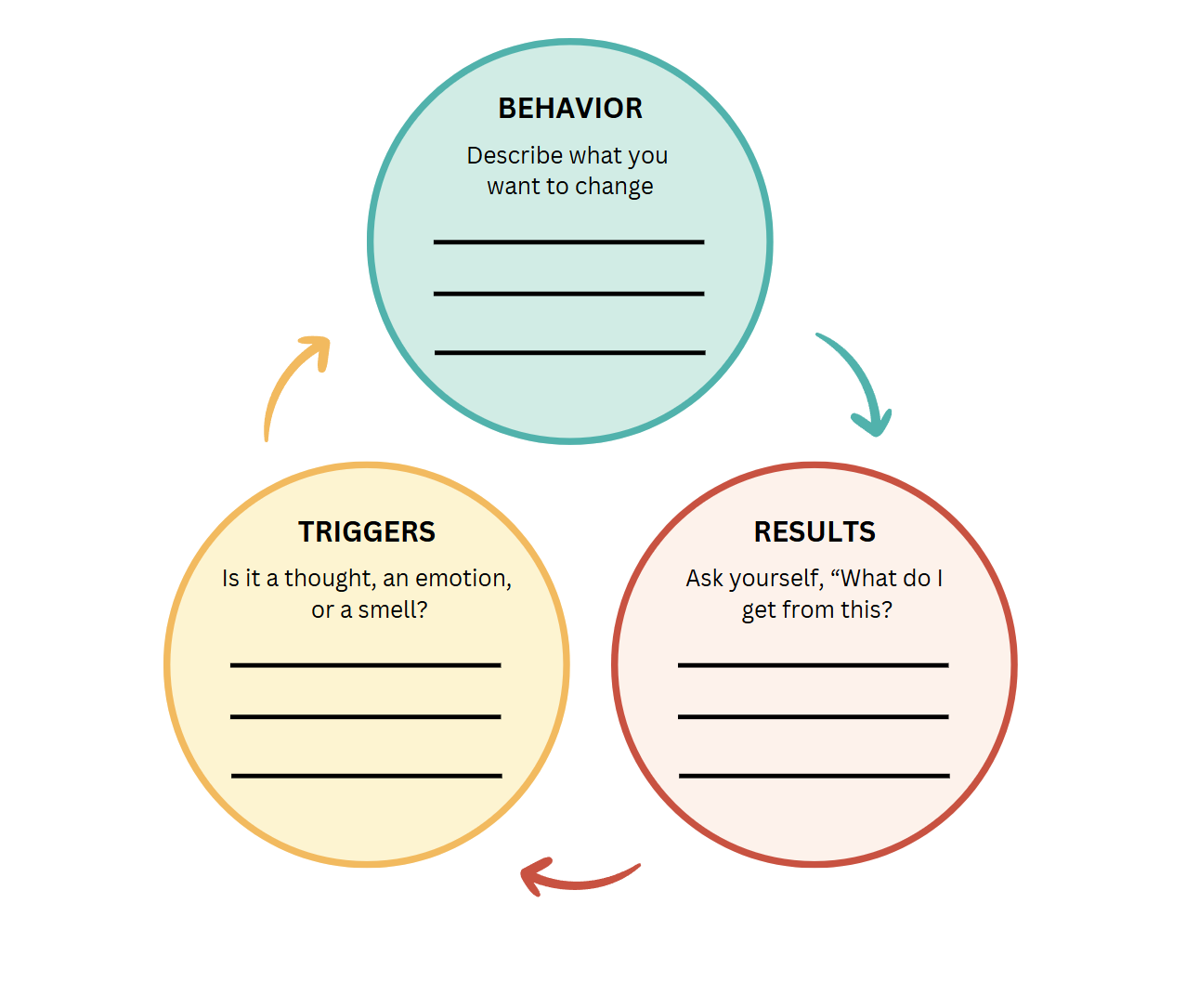
**

**PRACTICE NOTES**

**After Practice Journaling**

| What practice did you do? | How did your body feel during the practice? | What mood, feelings, and thoughts arose during the practice? | What are your thoughts or feelings now? |
| --- | --- | --- | --- |
| Monday |  |  |  |
| Tuesday |  |  |  |
| Wednesday |  |  |  |
| Thursday |  |  |  |
| Friday |  |  |  |
| Saturday |  |  |  |
| Sunday |  |  |  |

**Pleasant Experience Calendar**

| What experience occurred? | Did you notice a pleasant feeling when the experience happened? | What did your body feel in detail when the experience occurred? | What mood, feelings, and thoughts arose when the experience occurred? | What thoughts came to mind as you wrote this? |
| --- | --- | --- | --- | --- |
| Monday |  |  |  |  |
| Tuesday |  |  |  |  |
| Wednesday |  |  |  |  |
| Thursday |  |  |  |  |
| Friday |  |  |  |  |
| Saturday |  |  |  |  |
| Sunday |  |  |  |  |

**Unpleasant Experience Calendar**

| What experience occurred? | Did you notice an unpleasant feeling when the experience happened? | What did your body feel in detail when the experience occurred? | What mood, feelings, and thoughts arose when the experience occurred? | What thoughts came to mind as you wrote this? |
| --- | --- | --- | --- | --- |
| Monday |  |  |  |  |
| Tuesday |  |  |  |  |
| Wednesday |  |  |  |  |
| Thursday |  |  |  |  |
| Friday |  |  |  |  |
| Saturday |  |  |  |  |
| Sunday |  |  |  |  |

**Difficult Communication Calendar**

| Describe the communication that occurred. With whom? What was the topic? | How did the difficulty arise? | What did you want from the person/situation? What did you actually get? | What did the other person want? What did they actually get? | How did you feel during and after the situation? | Has the issue been resolved? If not, what is the resolution? |
| --- | --- | --- | --- | --- | --- |
| Monday |  |  |  |  |  |
| Tuesday |  |  |  |  |  |
| Wednesday |  |  |  |  |  |
| Thursday |  |  |  |  |  |
| Friday |  |  |  |  |  |
| Saturday |  |  |  |  |  |
| Sunday |  |  |  |  |  |

**Reference**

*4-Part Nonviolent Communication (NVC)—PuddleDancer Press*. (n.d.). Retrieved June 26, 2024, from https://www.nonviolentcommunication.com/learn-nonviolent-communication/4-part-nvc/

*8 Health Benefits of Sleep*. (2022, April 14). Sleep Foundation. https://www.sleepfoundation.org/how-sleep-works/benefits-of-sleep

Anuradha, R., Dutta, R., Raja, Jd., Sivaprakasam, P., & Patil, A. (2017). Stress and stressors among medical undergraduate students: A cross-sectional study in a private medical college in Tamil Nadu. *Indian Journal of Community Medicine*, *42*(4), 222. https://doi.org/10.4103/ijcm.IJCM_287_16

Arvant, A. Z., Wahyuningsih, S., & Dewi, S. Y. (2021). Factors Associated with Mental Distress Among Medical Students of Universitas Pembangunan Nasional Veteran Jakarta. *Review of Primary Care Practice and Education (Kajian Praktik Dan Pendidikan Layanan Primer)*, *4*(2), 4. https://doi.org/10.22146/rpcpe.62462

Black, D. S., & Slavich, G. M. (2016). Mindfulness meditation and the immune system: A systematic review of randomized controlled trials. *Annals of the New York Academy of Sciences*, *1373*(1), 13–24. https://doi.org/10.1111/nyas.12998

Brewer, J. (2021). *Unwinding anxiety: New science shows how to break the cycles of worry and fear to heal your mind*. Avery, an imprint of Penguin Random House.

Carrière, K., Khoury, B., Günak, M. M., & Knäuper, B. (2018). Mindfulness‐based interventions for weight loss: A systematic review and meta‐analysis. *Obesity Reviews*, *19*(2), 164–177. https://doi.org/10.1111/obr.12623

CDC. (2024a, May 10). *Benefits of Physical Activity*. Physical Activity Basics. https://www.cdc.gov/physical-activity-basics/benefits/index.html

CDC. (2024b, May 14). *Benefits of Healthy Eating for Adults*. Nutrition. https://www.cdc.gov/nutrition/php/resources/healthy-eating-benefits-for-adults.html

Chen, T.-L., Chang, S.-C., Hsieh, H.-F., Huang, C.-Y., Chuang, J.-H., & Wang, H.-H. (2020). Effects of mindfulness-based stress reduction on sleep quality and mental health for insomnia patients: A meta-analysis. *Journal of Psychosomatic Research*, *135*, 110144. https://doi.org/10.1016/j.jpsychores.2020.110144

Cipta, D. A., Wijovi, F., Melisa, L., Lili, R., Marcella, E., Tancherla, A., Siswanto, F. G., Adiya, D. A. K. L., Chen, S., Dermawan, G. J. C. I., Louis, M. I., Citraningtyas, T., & Molodynski, A. (2022). Burnout prevalence and degree among undergraduate medical students in Indonesia during 1 month of the COVID-19 pandemic: A cross-sectional descriptive survey. *International Journal of Social Psychiatry*, *68*(6), 1232–1237. https://doi.org/10.1177/00207640221116812

*Compassion vs. Empathy: What’s the Difference? - 2024*. (n.d.). MasterClass. Retrieved June 25, 2024, from https://www.masterclass.com/articles/compassion-vs-empathy

Curran, T., & Hill, A. P. (2019). Perfectionism is increasing over time: A meta-analysis of birth cohort differences from 1989 to 2016. *Psychological Bulletin*, *145*(4), 410–429. https://doi.org/10.1037/bul0000138

Cuttilan, A. N., Sayampanathan, A. A., & Ho, R. C.-M. (2016). Mental health issues amongst medical students in Asia: A systematic review [2000–2015]. *Annals of Translational Medicine*, *4*(4), 72.

Da Silva, C. C. G., Bolognani, C. V., Amorim, F. F., & Imoto, A. M. (2023). Effectiveness of training programs based on mindfulness in reducing psychological distress and promoting well-being in medical students: A systematic review and meta-analysis. *Systematic Reviews*, *12*(1), 79. https://doi.org/10.1186/s13643-023-02244-y

Ding, F., Wu, J., & Zhang, Y. (2023). Can mindfulness-based stress reduction relieve depressive symptoms? A systematic review and meta-analysis. *Journal of Pacific Rim Psychology*, *17*, 183449092211458. https://doi.org/10.1177/18344909221145814

Fahmi, A. Y., Dwidiyanti, M., & Wijayanti, D. Y. (2018). Effectiveness of Mindfulness on Decreasing Stress in Health Professional Students: A Systematic Review. *Holistic Nursing and Health Science*, *1*(1), 1. https://doi.org/10.14710/hnhs.1.1.2018.1-11

Finlay-Jones, A., Bluth, K., & Neff, K. (Eds.). (2023). *Handbook of Self-Compassion*. Springer International Publishing. https://doi.org/10.1007/978-3-031-22348-8

Godoy, L. D., Rossignoli, M. T., Delfino-Pereira, P., Garcia-Cairasco, N., & De Lima Umeoka, E. H. (2018). A Comprehensive Overview on Stress Neurobiology: Basic Concepts and Clinical Implications. *Frontiers in Behavioral Neuroscience*, *12*, 127. https://doi.org/10.3389/fnbeh.2018.00127

Gupta, S., Choudhury, S., Das, M., Mondol, A., & Pradhan, R. (2015). Factors causing stress among students of a Medical College in Kolkata, India. *Education for Health*, *28*(1), 92. https://doi.org/10.4103/1357-6283.161924

*Healthy diet*. (n.d.). Retrieved June 23, 2024, from https://www.who.int/news-room/fact-sheets/detail/healthy-diet

Hilton, L., Hempel, S., Ewing, B. A., Apaydin, E., Xenakis, L., Newberry, S., Colaiaco, B., Maher, A. R., Shanman, R. M., Sorbero, M. E., & Maglione, M. A. (2017). Mindfulness Meditation for Chronic Pain: Systematic Review and Meta-analysis. *Annals of Behavioral Medicine*, *51*(2), 199–213. https://doi.org/10.1007/s12160-016-9844-2

*How does mindfulness improve communication?* (2022, November 8). Mindfulness Supervision. https://mindfulness-supervision.org.uk/how-does-mindfulness-improve-communication/

*How Sleep Clears the Brain*. (2015, May 14). National Institutes of Health (NIH). https://www.nih.gov/news-events/nih-research-matters/how-sleep-clears-brain

Kabat-Zinn, J. (2013). *Full catastrophe living: Using the wisdom of your body and mind to face stress, pain, and illness*. Revised and updated edition. New York : Bantam Books, 2013. https://search.library.wisc.edu/catalog/9910217515502121

Khoury, B., Sharma, M., Rush, S. E., & Fournier, C. (2015). Mindfulness-based stress reduction for healthy individuals: A meta-analysis. *Journal of Psychosomatic Research*, *78*(6), 519–528. https://doi.org/10.1016/j.jpsychores.2015.03.009

Komariah, M., Ibrahim, K., Pahria, T., Rahayuwati, L., & Somantri, I. (2022). Effect of Mindfulness Breathing Meditation on Depression, Anxiety, and Stress: A Randomized Controlled Trial among University Students. *Healthcare*, *11*(1), 26. https://doi.org/10.3390/healthcare11010026

Lazarus, R. S., DeLongis, A., Folkman, S., & Gruen, R. (1985). Stress and Adaptational Outcomes. *American Psychologist*.

LeBouef, T., Yaker, Z., & Whited, L. (2024). Physiology, Autonomic Nervous System. In *StatPearls*. StatPearls Publishing. http://www.ncbi.nlm.nih.gov/books/NBK538516/

Lee, E. K. P., Yeung, N. C. Y., Xu, Z., Zhang, D., Yu, C.-P., & Wong, S. Y. S. (2020). Effect and Acceptability of Mindfulness-Based Stress Reduction Program on Patients With Elevated Blood Pressure or Hypertension: A Meta-Analysis of Randomized Controlled Trials. *Hypertension*, *76*(6), 1992–2001. https://doi.org/10.1161/HYPERTENSIONAHA.120.16160

Listiyandini, R. A., Andriani, A., Kinanthi, M. R., Callista, F., Syahnaz, H., Afsari, N., Ramadhan, M. R., Krisnamurthi, P. B. U., Moulds, M., Mahoney, A., & Newby, J. (2022). A culturally adapted internet-delivered mindfulness intervention for Indonesian university students’ distress: Overview of development and preliminary study. *Procedia Computer Science*, *206*, 206–209. https://doi.org/10.1016/j.procs.2022.09.099

Listiyandini, R. A., Andriani, A., Kusristanti, C., Moulds, M., Mahoney, A., & Newby, J. M. (2023). Culturally Adapting an Internet-Delivered Mindfulness Intervention for Indonesian University Students Experiencing Psychological Distress: Mixed Methods Study. *JMIR Formative Research*, *7*, e47126. https://doi.org/10.2196/47126

Loucks, E. B. (with Brewer, J.). (2022). *The mindful college student: How to succeed, boost well-being, and build the life you want at university and beyond*. New Harbinger Publications.

Lunn, J., Greene, D., Callaghan, T., & Egan, S. J. (2023). Associations between perfectionism and symptoms of anxiety, obsessive-compulsive disorder and depression in young people: A meta-analysis. *Cognitive Behaviour Therapy*, *52*(5), 460–487. https://doi.org/10.1080/16506073.2023.2211736

Manosso, L. M., Gasparini, C. R., Réus, G. Z., & Pavlovic, Z. M. (2022). Definitions and Concepts of Stress. In Z. M. Pavlovic (Ed.), *Glutamate and Neuropsychiatric Disorders* (pp. 27–63). Springer International Publishing. https://doi.org/10.1007/978-3-030-87480-3_2

Nair, M., Moss, N., Bashir, A., Garate, D., Thomas, D., Fu, S., Phu, D., & Pham, C. (2023). Mental health trends among medical students. *Baylor University Medical Center Proceedings*, *36*(3), 408–410. https://doi.org/10.1080/08998280.2023.2187207

*Perception: Definition, Examples, & Types*. (n.d.). The Berkeley Well-Being Institute. Retrieved June 26, 2024, from https://www.berkeleywellbeing.com/perception.html

Perry, R., Sciolla, A., Rea, M., Sandholdt, C., Jandrey, K., Rice, E., Yu, A., Griffin, E., & Wilkes, M. (2023). Modeling the social determinants of resilience in health professions students: Impact on psychological adjustment. *Advances in Health Sciences Education*, *28*(5), 1661–1677. https://doi.org/10.1007/s10459-023-10222-1

*Physical activity*. (n.d.). Retrieved June 23, 2024, from https://www.who.int/news-room/fact-sheets/detail/physical-activity

Polle, E., & Gair, J. (2021). Mindfulness-based stress reduction for medical students: A narrative review. *Canadian Medical Education Journal*. https://doi.org/10.36834/cmej.68406

*Practice: S.T.O.P.* (n.d.). Retrieved June 26, 2024, from http://accelerate.uofuhealth.utah.edu/resilience/practice-s-t-o-p

Primatanti, P. A., Turana, Y., Sukarya, W. S., Wiyanto, M., & Duarsa, A. B. S. (n.d.). Medical students’ mental health state during pandemic COVID-19 in Indonesia. *Bali Medical Journal*.

*Real-Life Benefits of Exercise and Physical Activity*. (2020, April 3). National Institute on Aging. https://www.nia.nih.gov/health/exercise-and-physical-activity/real-life-benefits-exercise-and-physical-activity

Sekhar, P., Tee, Q. X., Ashraf, G., Trinh, D., Shachar, J., Jiang, A., Hewitt, J., Green, S., & Turner, T. (2021). Mindfulness-based psychological interventions for improving mental well-being in medical students and junior doctors. *Cochrane Database of Systematic Reviews*, *2021*(12). https://doi.org/10.1002/14651858.CD013740.pub2

Shapiro, P., Lebeau, R., & Tobia, A. (2019). Mindfulness Meditation for Medical Students: A Student-Led Initiative to Expose Medical Students to Mindfulness Practices. *Medical Science Educator*, *29*(2), 439–451. https://doi.org/10.1007/s40670-019-00708-2

*Sleep & Insomnia Self-Help Resources—Information Sheets*. (n.d.). Retrieved June 24, 2024, from https://www.cci.health.wa.gov.au/Resources/Looking-After-Yourself/Sleep

Sperling, E. L., Hulett, J. M., Sherwin, L. B., Thompson, S., & Bettencourt, B. A. (2023). The effect of mindfulness interventions on stress in medical students: A systematic review and meta-analysis. *PLOS ONE*, *18*(10), e0286387. https://doi.org/10.1371/journal.pone.0286387

Tang, Y.-Y., Hölzel, B. K., & Posner, M. I. (2015). The neuroscience of mindfulness meditation. *Nature Reviews Neuroscience*, *16*(4), 213–225. https://doi.org/10.1038/nrn3916

thensf. (2020, October 1). How Much Sleep Do You Really Need? *National Sleep Foundation*. https://www.thensf.org/how-many-hours-of-sleep-do-you-really-need/

Ulrich-Lai, Y. M., & Herman, J. P. (2009). Neural regulation of endocrine and autonomic stress responses. *Nature Reviews Neuroscience*, *10*(6), 397–409. https://doi.org/10.1038/nrn2647

*Understanding Your Communication Style*. (n.d.). UMatter. Retrieved June 26, 2024, from https://umatter.princeton.edu/respect/tools/communication-styles

Van Gelderen, M. (2023). Using a comfort zone model and daily life situations to develop entrepreneurial competencies and an entrepreneurial mindset. *Frontiers in Psychology*, *14*, 1136707. https://doi.org/10.3389/fpsyg.2023.1136707

Wang, Z., Wu, P., Hou, Y., Guo, J., & Lin, C. (2024). The effects of mindfulness-based interventions on alleviating academic burnout in medical students: A systematic review and meta-analysis. *BMC Public Health*, *24*(1), 1414. https://doi.org/10.1186/s12889-024-18938-4

What is Self-Compassion? (n.d.). *Self-Compassion*. Retrieved June 25, 2024, from https://self-compassion.org/what-is-self-compassion/

Whitfield, T., Barnhofer, T., Acabchuk, R., Cohen, A., Lee, M., Schlosser, M., Arenaza-Urquijo, E. M., Böttcher, A., Britton, W., Coll-Padros, N., Collette, F., Chételat, G., Dautricourt, S., Demnitz-King, H., Dumais, T., Klimecki, O., Meiberth, D., Moulinet, I., Müller, T., … Marchant, N. L. (2022). The Effect of Mindfulness-based Programs on Cognitive Function in Adults: A Systematic Review and Meta-analysis. *Neuropsychology Review*, *32*(3), 677–702. https://doi.org/10.1007/s11065-021-09519-y
